# Effector immune cells in chronic lung allograft dysfunction: A systematic review

**DOI:** 10.1111/imm.13458

**Published:** 2022-03-01

**Authors:** Saskia Bos, Andrew J. Filby, Robin Vos, Andrew J. Fisher

**Affiliations:** ^1^ Newcastle University Translational and Clinical Research Institute Newcastle upon Tyne UK; ^2^ Institute of Transplantation The Newcastle Upon Tyne Hospital NHS Foundation Trust Newcastle upon Tyne UK; ^3^ Flow Cytometry Core and Innovation, Methodology and Application Research Theme Biosciences Institute Newcastle University Newcastle upon Tyne UK; ^4^ Department of CHROMETA Laboratory of Respiratory Diseases and Thoracic Surgery (BREATHE) KU Leuven Leuven Belgium; ^5^ Department of Respiratory Diseases University Hospitals Leuven Leuven Belgium

**Keywords:** adaptive immunity, chemokines, chronic lung allograft dysfunction, cytokines, immune cells, innate immunity, lung transplantation

## Abstract

Chronic lung allograft dysfunction (CLAD) remains the major barrier to long‐term survival after lung transplantation and improved insight into its underlying immunological mechanisms is critical to better understand the disease and to identify treatment targets. We systematically searched the electronic databases of PubMed and EMBASE for original research publications, published between January 2000 and April 2021, to comprehensively assess current evidence on effector immune cells in lung tissue and bronchoalveolar lavage fluid from lung transplant recipients with CLAD. Literature search revealed 1351 articles, 76 of which met the criteria for inclusion in our analysis. Our results illustrate significant complexity in both innate and adaptive immune cell responses in CLAD, along with presence of numerous immune cell products, including cytokines, chemokines and proteases associated with tissue remodelling. A clear link between neutrophils and eosinophils and CLAD incidence has been seen, in which eosinophils more specifically predisposed to restrictive allograft syndrome. The presence of cytotoxic and T‐helper cells in CLAD pathogenesis is well‐documented, although it is challenging to draw conclusions about their role in tissue processes from predominantly bronchoalveolar lavage data. In restrictive allograft syndrome, a more prominent humoral immune involvement with increased B cells, immunoglobulins and complement deposition is seen. Our evaluation of published studies over the last 20 years summarizes the complex multifactorial immunopathology of CLAD onset and progression. It highlights the phenotype of several key effector immune cells involved in CLAD pathogenesis, as well as the paucity of single cell resolution spatial studies in lung tissue from patients with CLAD.

**Abbreviations**
AMR: antibody‐mediated rejection
BALF: bronchoalveolar lavage fluid
BOS: bronchiolitis obliterans syndrome
CLAD: chronic lung allograft dysfunction
Ig: immunoglobulins
LTR: lung transplant recipients
MMP: matrix metalloproteinases
NK: natural killer
RAS: restrictive allograft syndrome
Tregs: T‐regulatory cells

## MULTIPLE FACES OF CHRONIC LUNG REJECTION

Lung transplantation is an established treatment option for patients with end‐stage lung diseases. However, long‐term success continues to be challenged by the development of chronic lung rejection, occurring in up to 50% of recipients within five years post‐transplant [1]. For a long time, obliterative bronchiolitis, and its clinical surrogate bronchiolitis obliterans syndrome (BOS), was the sole recognized manifestation of chronic lung rejection. Nowadays, the term chronic lung allograft dysfunction (CLAD) is used as an umbrella, which includes two main phenotypes, BOS and restrictive allograft syndrome (RAS), and a mixed phenotype [2, 3]. BOS is the best known and most common phenotype, in ~70% of CLAD patients, characterized by progressive airway obliteration leading to airflow obstruction [3]. RAS has more recently been acknowledged as another phenotype of CLAD, occurring in 20–30% of CLAD patients. It is characterized by interstitial fibrosis and distortion of lung architecture, a restrictive pulmonary function decline and persistent pleuroparenchymal abnormalities on computed tomography, and is associated with a poor median survival of only 1–2 years after diagnosis [3, 4]. Moreover, patients can switch from one phenotype (often BOS) to another (RAS/mixed) over time or present *de novo* with a mixed phenotype, characterized by mixed obstructive‐restrictive pulmonary function limitation and persistent parenchymal opacities [4]. The acknowledgement that there are different phenotypes suggests different underlying immunological mechanisms, although BOS and RAS also share commonalities such as the presence of obliterative bronchiolitis lesions in both entities, and areas of alveolar fibrosis in BOS. [5, 6, 7]

## COMPLEXITY OF THE UNDERLYING IMMUNOPATHOLOGY: A CHALLENGE

The exact immunopathological mechanisms leading to CLAD remain unclear, although multiple (immune) mechanisms are thought to contribute. Complex interactions between innate immune responses, alloreactive T, B, natural killer (NK) and dendritic cells, and subsequent adaptive immune mechanisms are considered to be fundamental [8]. Over the last decades, we have gained better understanding of the interactions between innate immunity, adaptive immunity and autoimmunity [9]. A better insight into all these processes is of utmost importance because, of all solid organ transplants, lung transplantation has the worst overall median survival of approximately 7 years [1, 10, 11, 12]. A better understanding of the mechanistic differences between CLAD phenotypes and involved pathways in the inflammatory and remodelling processes is crucial. On the one hand, this might help us to identify disease‐specific biomarkers that allow for early diagnosis, differentiation, and ideally predict CLAD development. On the other hand, it could lead to a personalized medicine approach through development of individualized therapies specific to each condition [13].

The primary objective of this systematic review is to comprehensively assess the phenotype of effector immune cells present in allograft tissue or bronchoalveolar lavage fluid (BALF) from lung transplant recipients (LTR) with CLAD. We postulate that most findings will be described in BOS patients, as the RAS/mixed phenotypes have only been recognized more recently. Since changes in effector immune cells at the peripheral blood level may contradict with what is detected at the allograft level, studies focusing on peripheral blood analyses were not included in this systematic review.

## METHODS

The systematic review was performed according to the Preferred Reporting Items for Systematic Reviews and Meta‐Analyses (PRISMA) 2020 guidelines [14].

### Search strategy and eligibility criteria

We conducted a systematic search on the electronic databases of PubMed and EMBASE using keywords related to immune cells and CLAD. Details on the search string can be found in Supplement 1, the last search was performed on 22 April 2021. The search was limited to publications from January 2000 onwards, English‐language articles, and articles with full‐text access. All titles and abstracts were reviewed thoroughly, followed by full‐text review if deemed eligible for inclusion. Further eligibility criteria were limited to original research articles, human data and analyses on lung tissue or BALF from patients with CLAD. We excluded studies that did not match the topic of interest and conference abstracts. In case of unclarity, inclusion was discussed until consensus was reached.

### Data extraction and synthesis

One reviewer (SB) screened all titles and abstracts and reviewed full‐text articles for study selection and collected data from the reports. If needed, data collection was discussed within the author team until consensus was reached. Relevant study characteristics including study design, sample size, CLAD phenotype, and type of analysis and its results were collected.

## RESULTS

### Literature search

**FIGURE 1:**
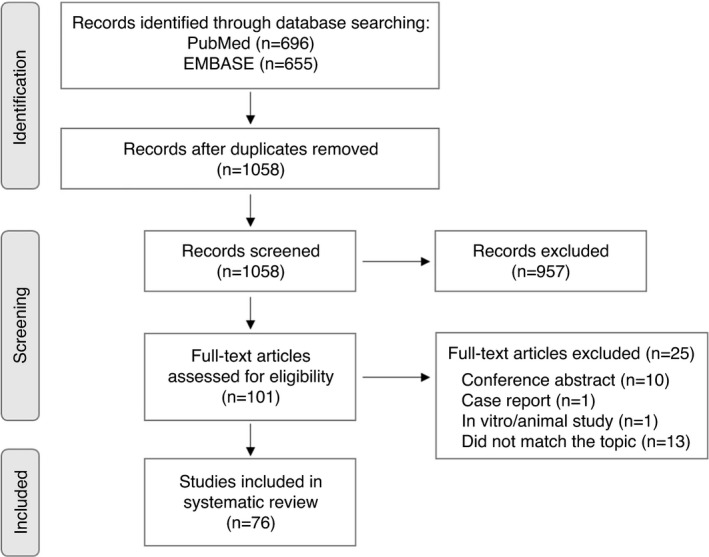
PRISMA 2020 flow diagram for systematic review

The systematic search revealed 1351 potentially relevant articles. After deleting duplicate records and primary screening, 101 articles were included for full‐text evaluation (Figure 1). Of these, 25 were excluded because they did not match the topic or study design. Characteristics of the included studies are presented in Supplement 1. Fifty‐one studies investigated BALF, 15 tissue analyses and 9 both tissue and BALF. Abbreviations for the factors analysed in BALF and tissue can be found in Table 1.

**TABLE 1:** Abbreviations for factors analysed in bronchoalveolar lavage fluid and tissue

C‐C motif chemokine ligand	CCL
C‐C motif chemokine receptor	CCR
Cluster of differentiation	CD
C‐X‐C‐L motif chemokine ligand	CXCL
Epithelial‐neutrophil activating peptide	ENA
Forkhead box P3	FoxP3
Granulocyte chemotactic protein	GCP
Human leucocyte antigen	HLA
Interferon gamma	IFN‐γ
Interferon gamma‐induced protein 10	IP‐10
Interferon–inducible T‐cell alpha chemo‐attractant	ITAC
Interleukin	IL
Interleukin 1 receptor antagonist	IL‐1RA
Macrophage inflammatory protein	MIP
Macrophage‐derived chemokine	MDC
Major histocompatibility complex	MHC
Matrix metalloproteinases	MMP
Monocyte chemo‐attractant protein	MCP
Monokine induced by interferon gamma	MIG
Pulmonary and activation‐regulated chemokine	PARC
Regulated upon activation, normal T‐cell expressed and secreted	RANTES
Thymus‐ and activation‐regulated chemokine	TARC
Tissue inhibitor of metalloproteinases	TIMP
Transforming growth factor beta	TGF‐β
Tumour necrosis factor alpha	TNF‐α

### Innate immune cells

#### Neutrophils

Numerous studies have described involvement of neutrophils in CLAD. Based on differential cell count, most studies found a significantly increased percentage in BALF in BOS compared to stable LTR [15, 16, 17, 18, 19, 20, 21, 22, 23, 24, 25, 26, 27], with also an increase in absolute numbers [15, 19, 21, 26, 27, 28, 29, 30]. Similar findings were found in studies that included RAS patients, with increased neutrophils in both BOS and RAS patients compared to stable LTR [13, 28, 31, 32, 33]. Few studies made a comparison with healthy controls and also noted increased neutrophils in stable LTR compared to them [15, 34, 35]. Upregulation of neutrophils (by neutrophil elastase staining) was also seen in BALF from RAS patients compared to stable LTR and BOS patients [36], and BOS patients versus stable LTR [36, 37].

Tissue analyses demonstrated increased neutrophils (by myeloperoxidase staining) in RAS explant lungs and airways of RAS and BOS patients compared to controls [38]. Zheng and colleagues demonstrated more neutrophils (by neutrophil elastase staining) in the airways in BOS as well as stable LTR compared to healthy controls, with no difference in the lung parenchyma (RAS was not yet identified at that time) [15]. The same group noted that airway wall neutrophilia, assessed by endobronchial biopsies, was similar to healthy controls at baseline, but increased over time in BOS patients [35].

Longitudinal analyses demonstrated increased BALF and/or endobronchial neutrophils at time of BOS diagnosis compared to pre‐BOS samples [25, 27, 30, 35]. Others already showed increased neutrophils in LTR who would go on to develop BOS compared to those who would remain stable [27, 39, 40]. Moreover, increased neutrophils correlated with increased BOS risk [39, 40]; more specifically, a BALF neutrophil percentage of ≥20% was a significant predictor for subsequent BOS ≥1 in a study by Neurohr et al. [40] Conversely, other studies could not demonstrate a difference in BALF neutrophils in future BOS or RAS patients compared to those who would remain stable [29, 33, 35].

Interestingly, Devouassoux et al. found no difference in neutrophil percentages in BOS stage 1 compared to stable LTR. In BOS stage 2, the increase of neutrophils occurred at BOS diagnosis, while in BOS stage 3, BALF neutrophilia preceded the diagnosis by 6 months [16]. Similarly, Heijink et al. found increased neutrophils in BALF from patients in BOS stage 1 who would progress to BOS stage 3 [24]. Finally, Vandermeulen et al. investigated a group of stable LTR with high (≥ 15%) versus low BALF neutrophil counts and found increased CLAD incidence and lower CLAD‐free and overall survival in the high‐neutrophil group [41]. The same group demonstrated that increased neutrophils (> 10%) in RAS patients correlated with worse graft survival [42].

#### Eosinophils

Data on eosinophils vary. In BOS patients, most studies found no elevated levels compared to stable LTR [13, 15, 17, 21, 23, 25, 26, 29, 30, 31, 34], while others noted an increase based on differential cell count [16, 22]. Scholma et al. found elevated numbers in the bronchial, but not alveolar, BALF fraction of future BOS patients, and elevated levels correlated with BOS risk [39]. In a study comparing stable LTR with high and low neutrophil counts, increased eosinophils were seen in the high‐neutrophil group [41]. In RAS patients, eosinophil percentages were higher than in stable LTR [28, 32, 33] or BOS patients [32]. More eosinophils (marked by EG2) were found in RAS explant lungs compared to controls and were primarily located in the lung parenchyma and around blood vessels [38].

BALF eosinophilia ≥2% correlated with CLAD and CLAD‐free survival, and the worst outcome was seen in LTR with high BALF and high blood (>8%) eosinophils [43]. Verleden et al. investigated the effects of episodes of eosinophilia in LTR and demonstrated that an episode of BALF eosinophilia (≥2%) correlated with worse CLAD‐free and overall survival, and predisposed to CLAD, mainly RAS but also BOS. The risk for CLAD and mortality was higher in case of multiple episodes of increased BALF eosinophilia [44]. The same group described a strong association between increased BALF eosinophils (≥2%) and survival after RAS diagnosis [42].

#### Macrophages

The percentage of BALF macrophages on differential cell count is often reported to be decreased in BOS patients compared to stable LTR, most likely secondary to an increase in other leucocytes, mainly neutrophils [13, 15, 16, 17, 18, 20, 21, 22, 23, 25, 26, 28, 31]. The same was true for patients with RAS compared to stable LTR [13, 28, 31, 32, 33]. Ward et al. found decreased expression of alveolar macrophage surface markers (CD11a, CD11b, CD11c, CD14 and HLA‐DR) in BOS and stable LTR compared to controls [34]. Most studies showed no difference in absolute macrophage numbers, although Vandermeulen et al. described an increase in BOS versus stable LTR and RAS patients [28]. On the other hand, on tissue analyses, more macrophages (CD68+) were found in RAS explant lungs compared to BOS and non‐transplant controls [38]. Zheng et al. described an increase on endobronchial biopsies in BOS and stable LTR over time compared to healthy controls [35].

#### Natural killer cells

Ward et al. found increased NK cells (CD56/CD16+) in both BOS and stable patients compared to healthy controls [34]. Other studies also noted increased BALF NK cells (CD56+) in BOS patients versus healthy controls, but not versus stable LTR [45, 46]. In addition, more NK cells were seen in small airway brushings in BOS patients compared to stable LTR and controls, with no changes in large airway brushings [45, 46]. In a study by Fildes et al., more NK cells (CD16+) were found on transbronchial biopsies from BOS patients than from stable patients [47]. Notably, Calabrese et al. showed that a certain subtype of NK cells, NKG2C+ NK cells, correlated with CLAD incidence [48]. Noteworthy, this impact on CLAD incidence may have been mediated by an effect on cytomegalovirus, as higher levels of NKG2C+ NK cells were found prior to and during cytomegalovirus infection, although the elevated risk remained after adjusting for cytomegalovirus serostatus and viraemia [48].

#### Mast cells

Few studies provide information on the presence of mast cells after lung transplantation. One study demonstrated an increase (marked by tryptase) in RAS explant lungs compared to non‐transplant controls. These mast cells were primarily located in the parenchyma and around blood vessels [38]. Another study differentiated between subtypes of mast cells and found an increase in total number of mast cells and subtype mast cell tryptase‐chymase over time after transplantation, with more mast cell tryptase in stable LTR >6 months post‐transplant compared to before. Moreover, they noted an increase in mast cell tryptase‐chymase in CLAD patients versus stable LTR [49].

#### Summary for innate immune cells

In summary for innate immune cells, we can state that neutrophils were generally elevated in BALF and lung tissue from BOS and RAS patients, and increased levels after transplantation correlated with increased CLAD incidence and lower CLAD‐free and overall survival. Higher levels of eosinophils were especially detected in RAS patients, while data varied in BOS studies. However, a clear correlation was again seen between elevated eosinophils and CLAD incidence (mainly RAS, but also BOS) and CLAD‐free survival.

It is too early to draw conclusions about changes in macrophages, NK cells or mast cells in BALF or lung tissue from CLAD patients. Usually, a decrease in BALF macrophage percentages was seen, secondary to an increase in other leucocytes, without a difference in absolute numbers; while one study showed higher numbers in RAS explant lungs compared to BOS. For NK cells, looking at different subtypes is promising.

### Adaptive immune cells

#### Dendritic cells

Dendritic cells form a link between innate and adaptive immunity. Leonard et al. found increased dendritic cells, marked by CD1a, MHC class II or RFD1, in BOS patients compared to stable LTR on both trans‐ and endobronchial biopsies. Markedly greater numbers were detected when using MHC class II expression and dendritic morphology than only CD1a as a marker [50]. A more recent study that included RAS patients, identified more dendritic cells (CD1a+) in the lung parenchyma in RAS explant lungs than in BOS or non‐transplant biopsies. More resident mucosal, langerin‐positive dendritic cells were present in the parenchyma in RAS compared to controls, but were decreased around the airways [38].

#### Lymphocytes

The majority of studies demonstrated no difference in BALF total lymphocytes based on differential cell count between CLAD patients and stable LTR [13, 15, 18, 20, 21, 22, 23, 25, 27, 29, 30, 31, 32, 33, 34, 35]. A few found elevated lymphocyte percentages or numbers in BOS [17, 24, 26, 28] or RAS [28] patients compared to stable, or in LTR with high versus low neutrophil counts [41]. Scholma et al. described increased lymphocyte numbers in the bronchial, but not alveolar, BALF fraction of future BOS patients compared to those who would remain stable, and elevated levels correlated with increased BOS risk [39]. In contrast, Zheng et al. found an almost significantly decreased lymphocyte percentage after BOS onset versus before (*p* = 0.057) [51]. With respect to tissue analyses, the same group found that the number of endobronchial lymphocytes was similar to healthy controls at baseline but increased over time in all LTR [35].

#### T‐lymphocytes

The proportion of BALF CD3+ lymphocytes was not significantly different between groups in some studies [20, 52, 53], while others showed an increase in BOS and stable LTR compared to healthy controls [34], or a decrease in BOS versus stable LTR [54] or healthy controls [45, 46, 54]. Various studies described increased CD8+ T cells with proportionally decreased CD4+ T cells in BOS versus stable LTR [55], or BOS and stable LTR versus healthy controls [34, 53]. Others found increased CD8+ and decreased CD4+ T cells in BOS patients versus controls, with increased CD8+ T cells in BOS versus stable LTR [45, 46, 54] and controls [54]. One study described opposing findings with increased CD4+ and decreased CD8+ T cells in BOS patients compared to stable LTR [20], while another study could not demonstrate a difference between groups [52].

A longitudinal study of Zheng et al. noted decreased BALF CD3+ T cells over time in BOS patients, and after BOS diagnosis compared to pre‐BOS samples. They could not demonstrate a longitudinal difference in CD4+ or CD8+ T cells [51]. Opposing findings were seen on endobronchial biopsies, with an increase in CD3+ and CD8+ T cells over time after transplantation, which was more pronounced in BOS patients. There was no significant difference after BOS diagnosis compared to before, but a trend was seen towards more CD8+ T‐cell infiltration in BOS patients than in stable LTR [51]. Another longitudinal study also demonstrated increased BALF CD8+ and decreased CD4+ T cells after BOS onset versus before [55].

Based on the varying data found in BALF regarding lymphocyte differential cell count and CD4/CD8 subtypes (i.e. stable vs. decreased vs. increased, as described above), it is difficult to make conclusions about underlying tissue processes. Devouassoux et al. found no difference in CD4+ or CD8+ T‐cells in transbronchial biopsies taken during the first year post‐transplant between patients who would remain stable and those who would develop BOS. However, there were more activated (CD25+ and CD69+) T cells in future BOS patients [56]. Vandermeulen et al. identified more cytotoxic T cells in RAS and BOS explant lungs than in non‐transplant controls [38]. Sato et al. also found more T cells in BOS explant lungs compared to non‐transplant controls, especially in areas of active obliterative and lymphocytic bronchiolitis compared to inactive obliterative bronchiolitis. These T cells were mainly effector memory T cells and were clustered into aggregates [57].

#### CD4+ T‐cell subsets

Several CD4+ helper T‐cell subtypes, including Th1, Th2 and T‐regulatory cells (Tregs), play a role in the pathogenesis of CLAD. Mamessier et al. demonstrated that there were more Th1 and Th2 cells in stable BOS than in non‐BOS patients, and more Th1 cells in evolving BOS than in stable LTR. Th2 activation was increased and Th1 activation was reduced in stable versus evolving BOS [58]. Several studies focused on Tregs, which are believed to have a role in regulating or suppressing effector T‐cell immune responses [52]. Bhorade et al. found less BALF FoxP3+ Tregs in BOS versus stable LTR. Furthermore, they identified more Tregs at one year post‐transplant in patients who would remain stable than those who would eventually develop BOS. More specifically, a threshold of 3·2% Tregs distinguished stable LTR from those developing BOS within the first two years post‐transplant. Additionally, CCL22, a chemokine involved in recruitment of Tregs, was also increased in the majority of stable patients, suggesting a potential mechanism by which these cells were attracted to the lung allograft [52]. Gregson et al. described no difference in total Tregs (CD25highFoxP3+) and CCR4 or CD103 subsets (essentially all Tregs were CCR4+ and CD103‐) in BALF from future BOS patients. On the other hand, increased CCR7+ Tregs protected against subsequent development of BOS. The CCR7‐ligand CCL21 correlated with CCR7+ Tregs and inversely with BOS, suggesting that this ligand might mediate recruitment of this Treg subset and downregulate alloimmunity [59]. Another study found more CD25highCD69‐ Tregs in stable and evolving BOS patients compared to stable LTR, with higher levels in stable versus evolving BOS patients [58]. Finally, Krustrup et al. noticed the highest number of FoxP3+ Tregs on transbronchial biopsies two weeks after transplantation. However, there was no effect of the number of FoxP3+ cells on BOS onset, nor did it predict time to BOS onset [60].

#### B‐lymphocytes and lymphoid follicles

Few studies focused on the presence of B cells in LTR and CLAD patients. A study investigating transbronchial biopsies during the first year post‐transplant noted increased CD20+ B cells in all LTR compared to non‐transplant controls [56]. More B cells were seen in areas of lymphocytic and active obliterative bronchiolitis than in areas of inactive obliterative bronchiolitis or healthy tissue [57]. Another study by Sato et al. demonstrated an increase in lymphoid aggregates in CLAD explant lungs versus non‐transplant controls, no further differentiation into BOS or RAS was made at that time [61]. Finally, a recent study investigating BOS and RAS explant lungs found more CD20+ B cells in both phenotypes compared to non‐transplant controls. Additionally, they found that RAS explant lungs contained more lymphoid follicles (‘tertiary lymphoid organs’) compared to BOS explant lungs and non‐transplant biopsies. These lymphoid follicles were predominantly localized around blood vessels and in the lung parenchyma [38].

#### Immunoglobulins

Deposition of immunoglobulins (Ig) has been described in the bronchial epithelium, basement membrane zone, bronchial wall microvasculature and chondrocytes in transbronchial biopsies from BOS patients compared to stable LTR and non‐transplant controls [62, 63]. A more recent study differentiated between BOS and RAS phenotypes, and found increased levels of IgG (total IgG and IgG1‐4) and IgM in BALF from RAS compared to BOS patients and stable LTR. IgA and IgE levels were also higher in RAS patients than in stable LTR, and higher total IgG and IgE levels were found in BOS versus stable LTR. Finally, increased IgG (total IgG, IgG1, IgG3 and IgG4) and IgM levels correlated with worse survival [28].

#### Summary for adaptive immune cells

With respect to adaptive immune cells, discordant data on BALF lymphocytes and CD4/CD8 subtypes have been reported, making it difficult to draw conclusions about underlying tissue processes. Most studies found no difference in total BALF lymphocytes, although a few found elevated levels in BOS and/or RAS patients. Data on lymphocyte subtypes varied: a majority found elevated CD8+ T cells with proportionally decreased CD4+ T cells in BOS patients, although others reported opposing findings or no differences. With regard to tissue analyses, findings were more consistent, with in general more cytotoxic T cells in CLAD patients (both RAS and BOS, especially in areas of active obliterative and lymphocytic bronchiolitis).

Surprisingly, few studies focused on the role of CD4+ T‐cell subtypes in CLAD. Both Th1 and Th2 cells were elevated in BOS compared to non‐BOS patients, with higher Th1 activity in evolving BOS and greater Th2 activation in stable BOS. Higher levels of Tregs were seen in stable LTR or stable compared to evolving BOS patients, and increased post‐transplant levels might protect against subsequent CLAD development.

Currently, there is limited published data on the presence of B cells in CLAD patients, but they showed more B cells in areas of lymphocytic and active obliterative bronchiolitis, Ig deposition and lymphoid aggregates, especially in RAS.

### Complement

Increased C3a was seen in BALF from BOS patients compared to non‐transplant controls [64]. Looking at both CLAD phenotypes, C4d [28, 65] and C1q [28] levels were elevated in RAS versus BOS and stable LTR, and correlated with mortality [28]. Two studies demonstrated lower levels of mannose‐binding lectin in BOS patients compared to stable LTR or controls [66]; and detection of mannose‐binding lectin at 3 and 6 months post‐transplant correlated with later development of BOS [67]. Deposition of mannose‐binding lectin was seen in the basement membrane and vasculature in BOS [68].

Magro et al. demonstrated increased C1q, C3, C4d, and C5b‐9 deposition in the bronchial epithelium, basement membrane zone, bronchial wall microvasculature and chondrocytes in BOS patients compared to stable LTR and non‐transplant controls [62]. Another study of the same group described bronchial wall deposition of C1q, C4d, and C5b‐9 in BOS patients, in which C1q deposition was the strongest predictor of BOS [63].

Intermediate and high levels of C3d correlated with BOS and bronchial wall or septal fibrosis, and all LTR with higher values of C3d within the septae or bronchial wall eventually developed BOS [69]. Similarly, Ngo et al. described that all LTR with high, multifocal C4d deposition developed CLAD [70]. Westall et al. found no association between early (<3 months post‐transplant) C3d or C4d deposition and BOS, but found significant intracapillary C3d/C4d deposition in all LTR with early BOS, along with light‐microscopic features suggestive of antibody‐mediated rejection (AMR) [71]. Ionescu et al. looked at C4d deposition in LTR with and without HLA antibodies and demonstrated that all patients with antibodies and subendothelial C4d deposition eventually developed BOS and/or graft loss [72]. Finally, downregulation of tissue complement‐regulatory proteins (CD55, CD46) has been described in BOS patients compared to non‐transplant controls [64].

In summary, various studies demonstrated increased complement levels and deposition in CLAD patients, and higher levels of complement deposition (e.g. C3d, C4d and C1q) predisposed to CLAD development.

### Matrix metalloproteinases

A summary of studies investigating matrix metalloproteinases (MMP) is provided in Table 2. [22, 24, 25, 28, 36, 37, 73, 74, 75] In general, most studies found an upregulation of MMP‐8 and/or MMP‐9 concentration and/or activity in BALF from CLAD patients compared to stable LTR. Neutrophils were the main source of MMP‐9 production [25], and MMP‐3 [24], MMP‐7 [24], MMP‐8 [22, 24], MMP‐9 [22, 24, 25, 37, 74] and TIMP‐1 [22] concentration and/or activity correlated with BALF neutrophils. Another study showed the airway epithelium itself as a direct source of MMP‐2 and MMP‐9 expression [74].

**TABLE 2:** BALF analyses of MMP in CLAD patients

	MMP‐1 concen‐tration	MMP‐1 activity	MMP‐2 concen‐tration	MMP‐2 activity	MMP‐3 concen‐tration	MMP‐3 activity	MMP‐7 concen‐tration	MMP‐7 activity	MMP‐8 concen‐tration	MMP‐8 activity	Pro‐MMP‐9	MMP‐9 concen‐tration	MMP‐9 activity	MMP‐12 concen‐tration	MMP‐12 activity	MMP‐13 concen‐tration	MMP‐13 activity	MMP‐8/TIMP‐1	MMP‐9/TIMP‐1	TIMP‐1	TIMP‐2	TIMP‐3	TIMP‐4	Comments
**BOS vs. stable LTR**																								
Hübner et al. [25]												↑	↑						↑*	↓				* elevated post‐BOS vs. pre‐BOS
Vandermeulen et al. [28]											↑	↑	↑											
Banerjee et al. [74]				↑									↑											Increased expression on bronchial/bronchiolar airway epithelium in BOS vs. stable LTR and healthy controls
Riise et al. [37]				=								↑	↑											
Hardison et al. [75]									↑	↑		↑	↑											Increased in post‐BOS vs. pre‐BOS
Verleden et al. [22]									↑			↑						↑	↑					Caused by a difference in protein concentrations in BOS patients with high BALF neutrophil counts with no differences between BOS patients with low neutrophil counts and stable LTR
Saito et al. [36]									↑															
Heijink et al. [24]	=	=	↑	=	↑	=	↑	=	↑	=		↑	=	=	=	=	=			↑	↑	=	=	No active MMPs in BOS patients, only MMP‐7 activity was detected in stable LTR. However, TIMP‐1‐bound MMP‐7, ‐8, and ‐9 and TIMP‐2‐bound MMP‐8 and ‐9 were increased in BOS, suggesting earlier activity of these MMPs
**Future BOS vs. stable LTR**																								
Ramirez et al. [73]													↑											
**RAS vs. stable LTR**																								
Vandermeulen et al. [28]											↑	↑	↑											
Saito et al. [36]									↑															

Note

Overview of studies showing BALF analyses of MMP in CLAD patients.

Abbreviations: ↑: increase; ↓: decrease; =: stable; BALF: bronchoalveolar lavage fluid; BOS: bronchiolitis obliterans syndrome; CLAD: chronic lung allograft dysfunction; LTR: lung transplant recipients; MMP: matrix metalloproteinases; RAS: restrictive allograft syndrome; TIMP: tissue inhibitor of metalloproteinases.

### Cytokines

#### IL‐8

With the exception of one study [76], increased BALF IL‐8 levels were found in BOS patients compared to stable LTR [13, 17, 18, 21, 24, 26, 27, 30, 33], or compared to stable LTR and healthy controls, and stable LTR compared to healthy controls [15]. Increased IL‐8 was also seen in stable LTR with high versus low neutrophil counts [41]. A correlation between IL‐8 and BALF neutrophils has been demonstrated in numerous studies [15, 22, 27, 35, 40], and also between BALF IL‐8 and endobronchial neutrophil numbers [35]. Interestingly, Verleden et al. found upregulation of IL‐8 in CLAD patients due to an upregulation in neutrophilic BOS with no difference between non‐neutrophilic BOS patients and stable LTR [22]. Longitudinal data showed increased levels after BOS diagnosis compared to pre‐BOS samples in many [27, 30, 35, 75], but not all [33], studies. Some studies demonstrated that IL‐8 was elevated in future BOS patients compared to those who would never develop BOS [27, 39, 40], and correlated with increased BOS risk [39], while Zheng et al. found persistently elevated levels in both future BOS patients and those who would remain stable compared to healthy controls [35]. Two recent studies included RAS patients and found no difference in IL‐8 levels between RAS and stable LTR [13, 33].

Regarding tissue analyses, increased IL‐8 expression was found on bronchial epithelial cells in a study by Elssner et al. [21] Finally, looking at donor lung biopsies, there was no difference in IL‐8 expression in future BOS or RAS patients compared to patients who would remain stable [77].

#### IL‐17

Several studies [17, 32, 78] demonstrated no differences in IL‐17 BALF levels between BOS and/or RAS patients and stable LTR, although elevated levels at 6–12 months post‐transplant were predictive of early BOS in a study by Fisichella et al. [17] Similarly, no difference was seen in stable LTR with high versus low neutrophils counts [41]. In a study looking at protein and mRNA levels, protein levels were under the detection level, but IL‐17 mRNA levels were increased in BOS patients compared to stable LTR [26]. Snell et al. looked at endobronchial presence of IL‐17, which was elevated early after transplant and subsequently decreased over time. There was a correlation with endobronchial CD8+ cells, but not with BALF IL‐8 levels, neutrophil percentages or BOS [79].

#### TGF‐β

Several studies described no differences in BALF TGF‐β levels between BOS and stable LTR [17, 29, 80, 81] or future BOS patients and those who would remain stable [73]. One study demonstrated that increased levels during the first 24h post‐transplant were associated with increased BOS risk, also after adjusting for primary graft dysfunction [82]. TGF‐β was expressed by bronchial epithelial cells, subepithelial mononuclear cells and alveolar macrophages, and TGF‐β receptor I by airway epithelium, peri‐airway and interstitial mononuclear cells, stromal cells and alveolar macrophages [82]. Elssner et al. found increased levels in BOS patients compared to stable LTR, but no increased TGF‐β expression on BALF or bronchial epithelial cells [21]. Hodge et al. noticed a longitudinal increase in BOS compared to pre‐BOS samples, but these data were only available in one patient [81]. Vanaudenaerde et al. differentiated between TGF‐β protein levels and mRNA and demonstrated no difference in protein levels, but an increase in TGF‐β mRNA in BOS patients compared to stable LTR [26]. On the other hand, Meloni et al. found a trend towards decreased TGF‐β in BOS compared to stable patients [18].

One recent study investigated both BOS and RAS patients and found increased levels in RAS compared to stable LTR. RAS patients with high TGF‐β levels had worse graft survival than those with low levels. On tissue analyses of RAS patients, TGF‐β1 was located in the (sub)pleural areas and patients with high TGF‐β1 expression had more local CD20+ B cells, CD4+ and CD8+ T cells, and CD68+ cells [83].

#### Other cytokines

Table 3 displays the main analyses of other cytokines in BALF in CLAD patients [13, 17, 18, 21, 22, 23, 26, 29, 30, 31, 32, 33, 39, 41, 73, 76, 78, 84, 85]. Additionally, in a study of donor lung biopsies, increased IL‐1β and IL‐6 expression were seen in future CLAD patients, and increased IL‐6 expression in pre‐implanted lungs of future BOS patients compared to RAS and stable LTR. There was a significant association between high IL‐6 expression and later BOS development [77].

**TABLE 3:** BALF analyses of cytokines in CLAD patients

	IL‐1β	IL‐1RA	IL‐2	IL‐4	IL‐5	IL‐6	IL‐7	IL‐9	IL‐10	IL‐12	IL‐13	IL‐15	IL‐16	IL‐23	TNF‐α	IFN‐γ	Comments
**BOS vs. stable LTR**																	
Fisichella et al. [17]	↑	=		=	=	=	=	↓		↓	=	=			=	=	Increased IL‐15, IL‐17, and TNF‐α 6‐12m post‐transplant was predictive of early‐onset BOS
Meloni et al. [18]									=	↓						=	Lower levels of IL‐12 were predictive of BOS
Vos et al. [76]						=											
Elssner et al. [21]									=						=		
Belperio et al. [29]	=	↑							=						=		Increased IL‐1RA preceded BOS onset
Laan et al. [23]													=				No difference at any time point
Vanaudenaerde et al. [26]	↑		↓			↑								↑			
Borthwick et al. [30]	↑														↑		Increased after BOS compared to before
Berastegui et al. [31]				=	=	=			=		=				=	↑	
Yang et al. [13]						=											
Keane et al. [84]																	
BOS vs. stable LTR fibrotic BOS vs. stable LTR treated BOS vs. stable LTR				= = =							↑ ↑ ↑						
Verleden et al. [22]																	IL‐1β correlated with BALF neutrophils
neutrophilic BOS vs. stable LTR neutrophilic vs. non‐neutr. BOS non‐neutrophilic BOS vs. stable	↑ ↑ =														= = =		
Verleden et al. [32] neutrophilic BOS vs. stable LTR non‐neutrophilic BOS vs. stable neutrophilic vs. non‐neutr. BOS	↑ = ↑	↑ = =	= = =	↑ = =			↑ = ↑	= = =	= = =		= = =						
Suwara et al. [33] ARAD vs. stable LTR PAN vs. stable LTR	↑ ↑	↑ ↑				= ↑									= ↑		Increased IL‐1α after BOS compared to pre‐BOS
**Future BOS vs. stable LTR**																	
Ramirez et al. [73]	=		=	=		=			=	=					=		
Scholma et al. [39]						↑											Increased IL‐6 correlated with increased BOS risk
**Stable LTR with high neutrophil count vs. low neutrophil count**																	
Vandermeulen et al. [41]	↑	↑	=	↑	↑		=	↑	↑	=	=	=			↑	=	Correlation between IL‐1β and IL‐4, IL‐8, CCL2, CCL3, CCL4, and CCL11. Correlating trend between IL‐1β and CLAD‐free survival (*p* = 0.084)
**RAS vs. stable LTR**																	
Suwara et al. [33]	=	=				↑									=		
Yang et al. [13]						=											
Berastegui et al. [31]																	
vs. stable LTR vs. BOS				= =	↑ ↑	= =			= =		= =				= =	↑ =	
Verleden et al. [32]																	IL‐6 was associated with survival after RAS diagnosis
vs. stable LTR vs. non‐neutrophilic BOS vs. neutrophilic BOS	↑ = =	↑ ↑ =	= = =	= = =	= = =	↑ ↑ ↑		= = =	= = =		= = =						
**Other**																	
Verleden et al. [85]	High IL‐6 levels first 24h post‐transplant correlated with better CLAD‐free and graft survival IL‐6 correlated with BALF neutrophils and IL‐8																
Neujahr et al. [78]	No correlation IL‐1RA, IL‐13 or IL‐17 during first year post‐transplant and future BOS or graft failure																

Note

Overview of studies showing BALF analyses of cytokines in CLAD patients.

↑: increase; ↓: decrease; =: stable; ARAD: azithromycin‐reversible allograft dysfunction; BALF: bronchoalveolar lavage fluid; BOS: bronchiolitis obliterans syndrome; CLAD: chronic lung allograft dysfunction; LTR: lung transplant recipients; PAN: persistent airway neutrophilia; RAS: restrictive allograft syndrome; other: see Table 1.

#### Summary for cytokines

Overall, numerous studies have examined BALF cytokines in CLAD patients and we can conclude that a correlation between IL‐8 and neutrophils is present, with elevated IL‐8 levels in BOS patients, especially neutrophilic BOS patients, and no change in RAS patients. Some studies reported increased TGF‐β levels in BOS patients, although several other studies failed to support this finding. Interestingly, a recent study documented increased levels in RAS patients that correlated with worse graft survival, perhaps suggesting a more prominent role for TGF‐β in this phenotype. Regarding other cytokines, levels were often not consistently different across groups, except that several studies reported increased IL‐1β and IL‐1RA in BOS patients, and some showed elevated IL‐6 levels in BOS and/or RAS patients. Finally, since mRNA and protein levels may differ, it is important to consider both methods of analysis.

### Chemokines

Table 4 provides an overview of BALF chemokines investigated in CLAD patients [13, 17, 18, 22, 27, 32, 39, 41, 78, 80, 86, 87, 88, 89, 90]. To summarize, several studies found elevated levels of chemokines CCL2/MCP‐1, CCL3/MIP‐1⍺, CCL4/MIP‐1β, CCL5/RANTES or CXCL10/IP‐10 in BOS and/or RAS patients, while others did not. With respect to tissue analysis, Sato et al. found increased CXCL12 in alveolar and airway epithelial cells and CCL21+ lymph vessels in CLAD explant lungs compared to non‐transplant controls [61].

**TABLE 4:** BALF analyses of chemokines in CLAD patients

	CCL2/MCP‐1	CCL3/MIP‐1α	CCL4/MIP‐1β	CCL5/RANTES	CCL7/MCP‐3	CCL11/eotaxin‐1	CCL17/TARC	CCL18/PARC	CCL19/MIP‐3β	CCL20/MIP‐3α	CCL22/MDC	CCL25/eotaxin‐3	CXCL5/ENA‐78	CXCL6/GCP‐2	CXCL9/MIG	CXCL10/IP‐10	CXCL11/ITAC	Comments
**BOS vs. stable LTR**																		
Fisichella et al. [17]	↓	=	=	↑		=										↑		
Meloni et al. [18]	↑			=														
Belperio et al. [87]															↑	↑	↑	Levels were not increased 4.5m before BOS onset
Belperio et al. [86]	↑																	Sources of CCL2 were airway epithelium and mononuclear cells
Reynaud et al. [27]	↑			↑														CCL2 correlated with BALF neutrophils and IL‐8
Verleden et al. [22] neutrophilic vs. non‐neutr. BOS	↑			↑														CCL2 and CCL5 correlated with BALF neutrophils
Verleden et al. [32] neutrophilic BOS vs. stable LTR neutrophilic vs. non‐neutr. BOS non‐neutr. BOS vs. stable LTR		↑ ↑ =	↑ = =	= = =	↑ = =			↓ ↓ =					= = =	= = =	= = =		= = =	
Sinclair et al. [80] BOS and stable LTR vs. healthy controls	↑																	
**Future BOS vs. future stable LTR**																		
Meloni et al. [88]		=	=				=		↑	↑	↑	=						Increased CCL19, CCL20 and CCL22 levels at 6m post‐transplant predicted BOS onset.
Scholma et al. [39]	=																	Increased CCL2 correlated with BOS risk.
Reynaud et al. [27]	↑			↑														
**Stable LTR with neutrophil high vs. low counts**																		
Vandermeulen et al. [41]	↑	↑	↑	↑		↑		=			=		=	=		↑		
**RAS vs. stable LTR**																		
Yang et al. [13]																=		Trend towards increased CXCL10 (*p*=.08).
Verleden et al. [32] vs. stable LTR vs. non‐neutrophilic BOS vs. neutrophilic BOS	↑ = =	↑ ↑ =	↑ ↑ =	= = =				= = ↑					= = =	= = =	= = =	↑ = =	= = =	CXCL10 and CXCL11 were associated with survival after RAS diagnosis.
**Other**																		
Meloni et al. [88]	No difference in CCR4, ‐6, or ‐7 expression but higher density of CCR6 in future BOS vs. stable LTR with increased CCR4 and ‐6 expression on CD68+ cells																	
Agostini et al. [89]	T‐cells expressing CXCR3 were found in areas of active obliterative bronchiolitis on transbronchial biopsies and BALF in BOS patients																	
Belperio et al. [87]	Prolonged elevation of CXCR3 ligands correlated with increased CLAD risk																	
Neujahr et al. [78]	Cumulative increased CXCL9 and CXCL10 during first year post‐transplant correlated with BOS and graft failure and preceded BOS onset by 3 and 9 months																	
Neujahr, Agostini, Shino et al. [78, 89, 90]	CXCL9, CXCL10 and CXCR3 were expressed by airway epithelial cells, mononuclear cells, and alveolar macrophages																	

Note

Overview of studies showing BALF analyses of chemokines in CLAD patients.

↑: increase; ↓: decrease; =: stable; BALF: bronchoalveolar lavage fluid; BOS: bronchiolitis obliterans syndrome; CLAD: chronic lung allograft dysfunction; LTR: lung transplant recipients; RAS: restrictive allograft syndrome; other: see Table 1.

## DISCUSSION

Post‐transplant airway and/or interstitial fibrosis results from a chronic immunological, inflammatory insult that leads to fibroproliferation and obliteration of distal airways and/or fibrosis of the lung parenchyma [27]. As presented here, multiple mechanisms are involved in CLAD (both BOS and RAS phenotypes), including allograft infiltration of innate immune cells, alloreactive T, B and NK cells, upregulation of numerous cytokines and chemokines, and matrix remodelling. Although BOS was first considered as a unique manifestation of chronic lung rejection, the identification of the RAS phenotype has changed our perception of this pathology [9]. As expected, less data are currently available on the specific mechanisms in RAS and the differences between RAS and BOS. After all, many studies predated the establishment of the RAS phenotype, although these chronic rejection groups probably also sometimes contained RAS patients.

Various findings overlap, such as the presence of neutrophils in BALF from patients with BOS and RAS, without differences between the two phenotypes [13, 28, 31, 32, 33]. On the other hand, the presence of eosinophils seemed more pronounced in RAS [32, 38]. Episodes of BALF eosinophilia predisposed to both CLAD phenotypes, but particularly RAS, with a strong correlation between increased BALF eosinophils and survival after RAS diagnosis [42, 44]. Theoretically, steroids inhibit eosinophil accumulation. However, increased eosinophilia in CLAD patients may indicate subtherapeutic steroid dosing or (relative) corticosteroid resistance as it was even present in patients with higher doses of corticosteroids, indicating that eosinophils might have an important role [41]. Eosinophilic granulocytes are able to release potent cytotoxic granule products, including proteins and cytokines, associated with cellular damage, and can regulate immune responses by attracting other immune cells via stored chemokines [39, 43]. Additionally, the release of eosinophilic cationic protein attracts fibroblasts and stimulates TGF‐β1 release, a known inducer of fibrosis [43, 44]. (Table 5) This makes us speculate about a possible role for eosinophils in the mechanism of tissue fibrosis in RAS [43].

**TABLE 5:** Function of innate immune cells

Cell type	Characteristics	Location	
Neutrophils [109]	Chemotaxis Phagocytosis Release of pro‐inflammatory cytokines, reactive oxygen species, hydrolytic enzymes and proteases,… Generation of neutrophil extracellular traps (NETosis) Epithelial‐to‐mesenchymal transition	Migration from circulation into tissue	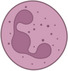
Eosinophils [110]	Release of cytokines, chemokines, reactive oxygen species, cytotoxic cationic granule proteins, enzymes,… Production of TGF‐β Epithelial‐to‐mesenchymal transition	Circulation in blood and migration into tissue	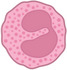
Macrophages [111]	Phagocytosis Antigen presentation Production of enzymes, complement proteins, and regulatory factors M1 (classically activated) macrophages: pro‐inflammatory cytokine release, bactericidal and phagocytic function, promotion of a local Th1 environment M2 (alternatively activated) macrophages: participation in type 2 immune responses, anti‐inflammatory cytokine release, tissue repair, production of TGF‐β	Tissue resident macrophages: alveolar macrophages, interstitial macrophages Migration from circulation into tissue	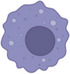
NK cells [112]	Activating and inhibitory receptors Cytolytic granule mediated cell apoptosis Antibody‐dependent cell‐mediated cytotoxicity Secretion of cytokines and chemokines Tumour cell surveillance Missing‐self (MHC I) recognition Clearance of senescent cells	Circulation in blood and migration into tissue	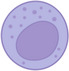
Mast cells [113]	Release of histamine, serine proteases (e.g. tryptase, chymase), cytokines, reactive oxygen species, and other mediators	Mucosal and epithelial tissues (including respiratory epithelium) Migration of mast cell progenitors upon antigen‐induced inflammation	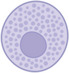
Dendritic cells [114]	Antigen presentation Release of pro‐inflammatory cytokines and chemokines	Present in lymphoid organs, blood, epithelial tissue (including lungs) Migration to lymph nodes upon activation	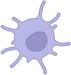

Note

Overview of some of the main general actions of innate immune cells. Images from BioRender.com.

Secondly, RAS has a more prominent humoral immune involvement, and the increase in B cells, immunoglobulins, and the presence of organized lymphoid follicles and complement is more specific in RAS [28, 38]. This raises the question whether there is a continuum between AMR and RAS [28]. AMR is usually caused by donor‐specific antibodies directed against donor human leucocyte antigens, leading to complement dependent and independent recruitment of immune cells leading to tissue injury and allograft dysfunction. AMR can present itself in a hyperacute (though currently rare due to improved antibody detection assays), acute or chronic form [91]. This has raised the thought whether RAS arises from a chronic form of AMR, although evidence supporting this paradigm is lacking. However, studies in this systematic review confirmed the higher presence of B‐cells, lymphoid follicles and immunoglobulins in RAS.

Besides the more pronounced presence of eosinophils and humoral immunity, not much is known about the differences at an immunopathological level between BOS and RAS. Reinvestigating old data in the light of our current knowledge would be useful, but presumably difficult to accomplish because not all details will be available, and we will therefore have to look for additional studies in the near future.

The same goes for the mixed phenotype. The reason why some patients transition from one phenotype to another remains poorly understood, although in some patients an episode of infection or AMR occurred between CLAD and mixed diagnosis [7]. Moreover, like in RAS *ab initio* patients, a higher number of circulating donor specific antibodies was seen in mixed phenotype patients, suggesting a role for humoral immunity. Additionally, similar histopathology findings were reported in patients that evolved from BOS to mixed and RAS *ab initio* patients, with survival rates comparable to RAS *ab initio* patients, suggesting a similar pathophysiology [7]. Regarding BALF analysis, Verleden et al. found no difference in total cell count, macrophages, neutrophils or lymphocytes between the mixed phenotype and RAS patients, but a higher percentage of eosinophils in the RAS group [7].

Given that a lot of risk factors (e.g. acute rejection, infection, non‐specific triggers of lung injury) are shared between BOS and RAS, combined with some similar findings in both entities (e.g. obliterative bronchiolitis lesions in RAS, areas of alveolar fibrosis in BOS) and the fact that patients can transition from one phenotype to another supports the hypothesis that BOS and RAS may be a continuum of the same disease [5, 6, 7]. Interestingly, there is considerable overlap between obliterative bronchiolitis after lung transplantation, after allogeneic hematopoietic stem cell transplantation and in clinical settings other than post‐transplant (e.g. post‐infectious) [92, 93]. Similarly, findings of alveolar and pleuroparenchymal fibroelastosis are not limited to RAS, but can also be found after allogeneic hematopoietic stem cell transplantation, drug exposure, radiation and occasionally idiopathic, suggesting a comparable immunological reaction to lung injury [92, 93, 94]. It therefore seems plausible that different causes of severe, repetitive or chronic lung injury can serve as a common denominator leading to inflammation and immune cell activation, and ultimately to pulmonary fibrosis, in which different clinical manifestations can be seen depending on the principal site of injury (bronchiolar/alveolar/vascular compartment) [5].

Traditionally, CLAD was thought to be primarily elicited by T‐cell immune responses, on which our currently used immunosuppressive regimens are based. However, we are nowadays aware of the multifactorial aetiology and contribution of many other factors, including pathologic B cells, innate immune cells and growth factors [8]. BALF profiles have been looked at in many studies and demonstrate involvement of neutrophils, eosinophils, NK cells, and possibly dendritic cells and mast cells. However, these results have proven to be not sensitive or specific enough to be relied on for accurate CLAD diagnosis [27]. Furthermore, the fact that not one specific innate immune cell is involved, but almost all types of innate immune cells, makes targeted therapy difficult.

Numerous studies illustrated neutrophilic inflammation as a driving force in this process, and BALF neutrophilia correlated with CLAD onset and severity [15, 16, 39, 40, 41]. Whether neutrophils were attracted to the airways because of infection and innate immune reaction, or as part of an alloreactive immune response to ‘non‐self’ antigens, they are potent effector cells [35]. Neutrophils contain strong pro‐inflammatory mediators, such as reactive oxygen metabolites, hydrolytic enzymes and proteases, which potentially induce tissue injury and extracellular matrix degradation [15]. An additional mechanism of neutrophil‐mediated cell injury is the formation of neutrophil extracellular traps and induction of epithelial‐to‐mesenchymal transition of lung epithelial cells [95, 96]. (Table 5) IL‐8 has been identified to account for a large portion of neutrophil chemotactic activity, and significantly higher percentages of neutrophils and IL‐8 levels were also detected in future BOS patients [15, 40]. IL‐17 might trigger IL‐8 and subsequent neutrophil chemotaxis [17, 97]. In contrast to this IL‐17‐driven neutrophilia, which is also the driver in azithromycin‐reversible allograft dysfunction [26, 98], IL‐1 (especially agonists IL‐1α and IL‐1β, and receptor antagonist IL‐1RA) can also be a source of persistent neutrophilia [33, 41]. Neutrophils play a key role not only in the onset of CLAD, but also in primary graft dysfunction for example, but given their important role in fighting infections, neutrophil actions cannot be completely negated [99].

The role of other innate cells in CLAD, for example dendritic, NK and mast cells, needs to be further clarified and some general immune functions of these cells are listed in Table 5. It is currently unclear whether these cells are actively involved in CLAD pathogenesis, or merely present because of more pronounced activation of and attraction by other cells. For example, increased dendritic cells in CLAD patients presumably reflect upregulation of expression of foreign allograft antigens [50]. Interestingly, in CLAD patients, peripheral blood NK cells were decreased but activated, while there was an increase in lung tissue, suggesting systemic activation and migration to the lung during CLAD [47]. This also highlights the importance of looking at the activation status, and not just the amount of immune cells present.

Thirdly, the precise involvement of macrophages in CLAD remains understudied and most of the included studies did not differentiate between macrophage subtypes. Macrophages are an essential component of the innate immune system, able to contribute to CLAD through pro‐inflammatory cytokine production, antigen processing and presentation, and tissue remodelling, but it is unclear whether they contribute solely by initiating immune responses or more specifically [100].

Finally, what has become less clear in these studies is the importance of different immune cell subtypes. Similar to T cells ranging from protective Tregs to cytotoxic T cells, more protective and more damaging NK cells exist, due to either activating or inhibitory actions through different receptors [101]. Calabrese and colleagues demonstrated that a specific subtype, NKG2C+ NK cells, correlated with CLAD incidence [48]. On the other hand, NK cells may promote graft tolerance through depletion of donor antigen‐presenting cells and alloreactive T cells via killer immunoglobulin‐like receptors [101]. The same probably also applies to eosinophils, where it has recently been illustrated in animal models that eosinophils can downregulate alloimmunity. These immunosuppressive effects are presumably exerted by a different subtype of eosinophils [102].

We deliberately excluded studies with peripheral blood analyses, as these findings do not always reflect what is happening at a tissue level in the allograft. For example, immune cells can be attracted from the systemic circulation into the allograft (and thus be normal or decreased in serum while elevated in the allograft). Furthermore, even lung tissue and BALF analyses can be contradictory, which we saw especially in the lymphocytes and their subsets, where the data were not always consistent with more consistent findings in tissue, highlighting the importance of tissue analyses.

The actions of effector T and B cells remain crucial in the pathogenesis of CLAD, and immunological reactions are regulated by different subsets of T cells, ranging from cytolytic activity (CD8+ T cells, Th1 cells), activation of innate and adaptive immune cells, to propagating (pro‐inflammatory/profibrotic cytokine release from Th1 and some Th2 cells) or dampening inflammation (Tregs, anti‐inflammatory cytokine release from Th2 cells) [52, 58, 103]. Overall, increased cytotoxic T cells were present in CLAD patients, especially in areas of ongoing fibrosis. It is surprising how few BALF and/or tissue studies were found that focused on the effects of these subtypes in CLAD. In future research, it will be important to look at more detail not only at the presence of these adaptive immune cells but also their activation status as well as the exact roles of different subtypes, including effector memory T and B cells, tissue resident cells, and γδ‐T cells in the onset of CLAD.

The adaptive immune response relies on the ability of T and B cells to undergo extensive cell division and clonal expansion to generate an adequate immune response to antigen exposure. Therefore, in contrast to many other somatic cell lineages, T and B cells express high levels of telomerase activity at regulated stages of development and upon activation of mature cells. Telomeres and telomerase play a critical role in the regulation of the replicative lifespan of cells. Briefly, telomeres are repetitive nucleotide sequences located on the terminal region of chromosomes that protect the integrity of chromosomes during cell replication. Telomere length decreases with cellular ageing and biologic stressors, but excessive shortening triggers cellular senescence or apoptosis. Telomerase is an enzyme that synthesizes telomeres and compensates for telomere loss that occurs with cell division [104, 105, 106, 107]. Consequently, individuals with short telomeres (whether or not caused by mutations in the telomerase maintenance mechanism) are more susceptible to a range of premature organ dysfunctions such as pulmonary fibrosis. After lung transplantation, it has been shown that these patients had a higher incidence of clinically significant leukopenia and CLAD, with decreased CLAD‐free survival [106, 107].

Finally, the actions of many immune cells rely on the presence of cytokines and chemokines to activate and direct them into the allograft [31]. Of the chemokines found to be upregulated in CLAD, CCL3/MIP‐1α, CCL5/RANTES, CCL7/MCP‐3 and CCL11/eotaxin are known to attract eosinophils, while most chemokines are able to recruit macrophages and/or T cells [32, 41].

The three IFN‐γ‐induced CXCR3 ligands, CXCL9/MIG, CXCL10/IP‐10 and CXCL11/ITAC, have been shown to be important in CLAD [78, 87, 88, 89]. Persistent expression leads to ongoing peribronchial/‐bronchiolar leucocyte infiltration, which eventually promotes fibrotic remodelling, and blockade of CXCR3 was associated with a significant reduction in intra‐graft mononuclear cell infiltration [87, 88]. Similar results were seen with CCL2/MCP‐1, a potent mononuclear phagocyte chemo‐attractant. CCL2 also correlated with neutrophils and IL‐8, demonstrating distinct mechanisms by which a specific receptor/chemokine biological axis may be involved in the pathogenesis of BOS and RAS [18, 22, 27, 32, 86, 88].

The role of CCL19/MIP‐3β has not been widely studied, but CCR7, the receptor for CCL19 and CCL21, is involved in migration of central memory T cells and mature dendritic cells, and maturation and differentiation of T cells [88]. In addition, a role in tissue repair mechanisms has been implicated as CCR7 is expressed on peripheral blood fibrocytes, airway smooth muscle, and fibroblasts. The CCR7/CCL19 axis seemed to play a role in airway smooth muscle hyperplasia in asthmatics and CCR7 was also expressed on fibroblasts in fibrotic areas of idiopathic pulmonary fibrosis patients [88]. Altogether, a possible involvement of CCL19/CCR7 interaction in the fibroproliferative process of CLAD has been suggested [88].

Several limitations of the studies included in this systematic review need to be addressed, in addition to the fact that most focused on the BOS phenotype. Most studies had a cross‐sectional study design and a small study population. Different types of analyses and techniques have been used, making an adequate comparison difficult, and findings were often inconsistent. The impact of other factors, such as airway infection or colonization, is not discussed in this review, although many studies took this into account or excluded these patients.

Finally, this systematic review focused on immune cells and cytokines and chemokines involved in CLAD pathogenesis, but we know CLAD is a much more complex pathology involving many other factors, such as different types of antibodies and fibrotic growth factors. Also, emerging evidence underscored significant interactions between autoimmunity and alloimmunity after transplantation, with involvement of Th17 cells and IL‐17, and lung‐associated self‐antigens (e.g. collagen V, K‐alpha 1 tubulin) [108].

## FUTURE RESEARCH DIRECTIONS

Based on these findings, future research should include studies to address the following:

- Specific mechanistical differences between CLAD phenotypes, especially BOS versus RAS;
- Use of single cell and spatial studies in lung tissue;
- Disease‐specific BALF biomarkers for timely diagnosis and endo/phenotyping of CLAD;
- Identifying specific immune cells or (profibrotic) pathways in the pathogenesis of CLAD which are targetable for treatment;
- Use of BALF gene expression profiling to identify LTR at risk for acute rejection and/or CLAD;
- Developing immunosuppressive drugs specifically targeting certain subtypes of T and B cells, upregulating Tregs, and/or modulating other immune cells involved in CLAD pathogenesis.

## CONFLICT OF INTEREST

None of the authors of this manuscript have any conflicts of interest to disclose in relation to this manuscript. The authors confirm that the work described has not been published previously, that it is not under consideration for publication elsewhere, that its publication is approved by all authors and tacitly or explicitly by the responsible authorities where the work was carried out, and that, if accepted, it will not be published elsewhere in the same form in English or in any other language, without the written consent of the copyright holder.

The data that support the findings of this study are available on request from the corresponding author. All authors contributed in an important manner to the study design, data collection and analysis, or writing of the paper according to the guidelines of the International Committee of Medical Journal Editors (ICMJE). All authors have read and approved the manuscript, all authors take responsibility for the manuscript, and the submitting author has permission from all authors to submit the manuscript on their behalf.

## AUTHOR CONTRIBUTION

SB performed research, collected data, wrote the manuscript. AF and RV critically revised the manuscript. AJF co‐ordinated and designed the research, critically revised the manuscript.

## Supporting information

Supplementary Material
