# Supplementary material for: Effector immune cells in chronic lung allograft dysfunction: A systematic review
Source: Immunology. 2022 Mar 1;166(1):17–37. doi: 10.1111/imm.13458 (PMC9426626; doi:10.1111/imm.13458)
Supplement: Supplementary file 1 — Supplementary Material [file IMM-166-17-s001.docx]

**Appendix 1: search string**

PubMed

("Leukocytes"[Mesh] OR "Macrophages"[Mesh] OR "complement" OR "mannose-binding lectin" OR "Matrix Metalloproteinases"[Mesh] OR "Cytokines"[Mesh] OR "neutrophilia" OR "eosinophilia" OR "Bronchoalveolar Lavage"[Mesh] OR "flow cytometry" OR "Histology"[Mesh] OR "lung biopsy" OR "transbronchial biopsy" OR "lung tissue") AND ("chronic lung allograft" OR "CLAD" OR "chronic lung rejection" OR "restrictive allograft" OR "rCLAD" OR "bronchiolitis obliterans syndrome" OR "obliterative bronchiolitis")

Filter: Publication start date: 01 Jan 2000, language: English, text availability: full text.

EMBASE

('matrix metalloproteinase'/exp OR 'mannose binding lectin' OR 'leukocyte'/exp OR 'macrophage'/exp OR 'cytokine'/exp OR 'complement'/exp OR 'neutrophilia' OR 'eosinophilia' OR 'bronchoalveolar lavage fluid'/exp OR 'histology'/exp OR 'flow cytometry'/exp OR 'lung biopsy'/exp OR 'lung tissue') AND ('chronic lung allograft dysfunction'/exp OR 'bronchiolitis obliterans syndrome'/exp OR 'restrictive allograft syndrome'/exp OR 'rclad' OR 'obliterative bronchiolitis' OR 'chronic lung rejection') AND [english]/lim AND [2000-2021]/py

**Appendix Table 1: overview of included articles**

| **Abbreviations used in table** | | | |
| --- | --- | --- | --- |
| **Ab**  **ACR**  **AMR**  **AR**  **ARAD**  **BALF**  **BB**  **BOS**  **CCL**  **CCR**  **CD**  **CLAD**  **CMV**  **CRP**  **CXCL**  **DAD**  **DSA**  **EBB**  **fBOS**  **FoxP3**  **GCP**  **GM-CSF**  **HLA**  **IF**  **IFN-γ**  **Ig**  **IHC**  **IL** | antibodies  acute cellular rejection  antibody-mediated rejection  acute rejection  azithromycin-reversible allograft dysfunction  bronchoalveolar lavage fluid  bronchial brushing  bronchiolitis obliterans syndrome  C-C motif chemokine ligand  C-C motif chemokine receptor  cluster of differentiation  chronic lung allograft dysfunction  cytomegalovirus  C-reactive protein  C-X-C motif chemokine ligand  diffuse alveolar damage  donor-specific antibodies  endobronchial biopsy  fibrotic bronchiolitis obliterans syndrome  forkhead box P3  granulocyte chemotactic protein  granulocyte-macrophage colony-stimulating factor  human leukocyte antigen  immunofluorescence  interferon gamma  immunoglobulin  immunohistochemistry  interleukin | **IL-1RA**  **IP-10**  **ITAC**  **LB**  **LTR**  **LTx**  **MBL**  **MCP**  **MDC**  **MHC**  **MIG**  **MIP**  **MMP**  **MPO**  **NK**  **NRAD**  **OB**  **PGD**  **RANTES**  **RAS**  **TARC**  **TBB**  **TGF-β**  **TIMP**  **TNF-α**  **Tregs**  **VEGF** | interleukin 1 receptor antagonist  interferon gamma-induced protein 10  interferon–inducible T-cell alpha  chemoattractant  lymphocytic bronchiolitis  lung transplant recipient  lung transplantation  mannose-binding lectin  monocyte chemoattractant protein  macrophage-derived chemokine  major histocompatibility complex  monokine induced by interferon gamma  macrophage inflammatory protein  matrix metalloproteinases  myeloperoxidase  natural killer  neutrophilic reversible allograft dysfunction  obliterative bronchiolitis  primary graft dysfunction  regulated upon activation, normal T-cell expressed and secreted  restrictive allograft syndrome  thymus- and activation-regulated chemokine  transbronchial biopsy  transforming growth factor beta  tissue inhibitor of metalloproteinases  tumor necrosis factor alpha  regulatory T-cells  vascular endothelial growth factor |

| **Author** | **Study design** | **Population** | **BALF/tissue** | **Analysis** | **Results** |
| --- | --- | --- | --- | --- | --- |
| Agostini *et al*, 2001^1^ | Retrospective | 24 LTR (3 BOS and 8 TBB with AR) | BALF  TBB | CXCL10/IP-10, CXCR3  In vitro chemotaxis | BALF: T-cells expressed CXCR3 and IFN-γ during AR and BOS. TBB: areas of AR and active OB were infiltrated by T-cells expressing CXCR3. TTB and BALF: high expression of CXCL10 by macrophages and occasionally by epithelial cells in AR and BOS.  Higher expression of CXCR3 and IFN-γ on BALF T-cells and CXCR3 on TBB T-cells in higher grade AR than lower grade (p<.01). |
| Banerjee *et al*, 2011^2^ | Prospective | 8 BOS  18 stable LTR  10 healthy controls | BALF  BB | MMP-2, MMP-9, TIMP-1, TIMP-2 | Increased BALF neutrophils (%) in BOS vs stable LTR (p=.08).  Increased MMP-2 and -9 activity in BALF and bronchial and bronchiolar airway epithelium expression in BOS vs stable LTR and controls (all p≤0.01). Airway epithelium was a direct source of MMP-2 and -9 expression in BOS patients.  Increased MMP-9/TIMP-1 and MMP-2/TIMP-2 ratio in BOS vs stable LTR (both p≤0.01). No difference in TIMP-1 or -2 expression. No difference in MMP-2 and MMP-9 expression between small and large airways.  No correlation between BALF neutrophils and MMP-2 or -9 expression, correlation between BALF neutrophils and MMP-2 and -9 activity (p≤0.01). |
| Banga *et al*, 2016^3^ | Retrospective  Longitudinal | 5 CLAD | TBB | Mast cell (MC), MC-tryptase (MC-t), MC-tryptase/chymase (MC-tc)  determined during early stable post-LTx (< 6m), late stable post-LTx (> 6m), ACR, and CLAD | MC (#) and MC-tc (#) increased over time (both p<.01).  Increased MC-tc (#) in CLAD vs others (all p<.05), increased MC-tc/MC-t ratio in CLAD vs others (all p<.001).  Increased MC-t (#) in late stable vs early stable (p=.04). |
| Belperio *et al*, 2001^4^ | Prospective  Cross-sectional | 20 BOS  27 AR  30 stable LTR | BALF | CCL2/MCP-1  In vitro chemotaxis, role of MCP-1 in murine model | Increased CCL2 in BOS vs stable LTR, and AR vs stable LTR, with more mononuclear cell chemotaxis (all p≤.01). Sources of CCL2 were airway epithelium and mononuclear cells. |
| Belperio *et al*, 2002^5^ | Prospective  Longitudinal | 108 LTR | BALF | CXCL9/MIG, CXCL10/IP-10, CXCL11/ITAC  Role of CXCR3 in murine model | Increased CXCL9, CXCL10, and CXCL11 in BOS and AR vs stable LTR (all p<.05). Elevated levels were predictive of acute or chronic rejection. No increase of CXCL9, CXCL10, and CXCL11 at a mean of 4.5 months before BOS onset. |
| Belperio *et al*, 2002^6^ | Prospective  Longitudinal | 22 BOS  33 AR  30 stable LTR | BALF | IL-1RA, IL-1β, IL-10, TGF-β, TNF-α | Increased neutrophils (#) in BOS vs AR and stable LTR (both p<.05), no difference in future BOS.  Increased IL-1RA in BOS vs AR and stable LTR (both p<.05), and this preceded BOS onset.  No difference in IL-1β, IL-10, TGF-β, or TNF-α. |
| Berastegui *et al*, 2017^7^ | Retrospective  Cross-sectional | 15 BOS  7 RAS  29 stable LTR | BALF | Differential cell count, IL-4, IL-5, IL-6, IL-10, IL-13, TNF-α, IFN-γ  GM-CSF | Increased neutrophils (%) and decreased macrophages (%) in BOS and RAS vs stable LTR (all p≤0.01).  Increased IFN-γ in BOS and RAS vs stable LTR, increased IL-5 in RAS vs BOS and stable LTR (all p<.05). |
| Bhorade *et al*, 2010^8^ | Prospective  Longitudinal | 20 LTR (6 developed BOS) | BALF | CD3, CD4, CD8, CD25, FoxP3, CCL17, CCL22/MDC  Blood analyses | Decreased CD4+FoxP3+ cells (%) and CCL22 in BOS vs stable LTR (both p<.05), no difference in CCL17.  Increased CD4+FoxP3+ cells (%) at 1y in LTR who would remain stable vs future BOS (p=.017). Threshold of 3.2% CD4+FoxP3+ distinguished stable LTR from those developing BOS within first 2y post-LTx.  No difference between BOS and stable LTR CD3+, CD4+, CD8+ T-cells in BALF.  During AR, more BALF CD4+FoxP3+ cells (%) in LTR who did not progress to BOS (p=.002). More CD3+ T-cells in blood than in BALF in BOS and stable LTR. No difference in blood CD4+FoxP3+ cells, CD3+, CD4+, CD8+ T-cells in BOS vs stable LTR. More CD4+FoxP3+ cells (%) in BALF vs blood in stable LTR at 1y. |
| Borthwick *et al*, 2013^9^ | Retrospective  Longitudinal | 52 LTR (26 developed BOS) | BALF | Differential cell count, IL-1β, IL-8, TNF-α  In vitro analyses | Increased total cell count and neutrophils (#) in < 3 mos. prior to BOS vs > 3 mos. prior to BOS and stable LTR (both p<.01). No difference in macrophages, eosinophils, lymphocytes (#).  Increased IL-1β, IL-8, and TNF-α in < 3 mos. prior to BOS vs > 3 mos. prior to BOS (> 3 mos.) and stable LTR (all p<.001).  Increased total cell count, neutrophils, TNF-α, IL-1β, and IL-8 in *P. aeruginosa* culture positive LTR vs culture negative LTR (all p<.05). |
| Budd *et al*, 2012^10^ | Retrospective  Cross-sectional | 8 BOS explant lungs  6 at time of implantation  6 DAD in non-LTx controls | Biopsies | MBL  Blood MBL, C3, C4 | MBL localized to vasculature and basement membrane during cold ischemia and BOS.  Increased plasma MBL in BOS vs stable LTR and in LTR < 5 years vs > 5 years post-LTx (all p<.05). Increased plasma C3 in BOS vs stable LTR, increased plasma C4 in > 5 years post-LTx BOS vs stable LTR (all p<.05). |
| Calabrese *et al*, 2019^11^ | Prospective | 130 LTR | BALF | NK cells (NKG2A, NKG2C, KIR2D, KIR3D, KIR3DL1, CD56, CD3, CD45)  Blood analyses (n=40 LTR) | Increased NKG2C+ NK cells correlated with CLAD.  7.2% of the NK cells were NKG2C+. NKG2C+ NK cells were more mature and proliferative than NKG2C- NK cells (all p<.001).  Increased NKG2C+ NK cells in LTR with high CMV viraemia (p=.0001).  No difference or correlation between BALF and blood NK cells, no difference but positive correlation between BALF and blood NKG2C+ NK cells. |
| Carroll *et al*, 2011^12^ | Retrospective  Longitudinal | 37 LTR | BALF | MBL  Blood analyses | Detection of MBL in BALF at 3 and 6m post-LTx correlated with later development of BOS (both p<.05).  Blood MBL correlated with MBL-mediated C4d deposition (p<.001).  Increased blood MBL at 3, 6, and 12m post-LTx vs pre-LTx (all p<.05). LTR who developed BOS or died had higher blood MBL at 6 and 12m post-LTx vs stable LTR (p<.05).  No correlation blood MBL and PGD, ACR, sepsis, or micro-organism isolation (p<.05). Low pre-LTx blood MBL correlated with CMV reactivation (p=.04). |
| DerHovanessian *et al*, 2016^13^ | Retrospective  Cross-sectional | 75 LTR  5 surgical biopsy | BALF < 24h post-LTx  Surgical biopsy | BALF: TGF-β, procollagen  Biopsy: TGF-β and TGF-β receptor I expression (TGF-βRI) | Increased BALF TGF-β and procollagen < 24h post-LTx were associated with increased BOS risk (both p<.05), TGF-β remained significant after adjusting for PGD (p=.01).  Correlation between TGF-β and procollagen (p<.001), no longer significant in multivariable models after adjustment for PGD severity.  TGF-β expression by bronchial epithelial cells, subepithelial infiltrating mononuclear cells, alveolar macrophages; TGF-βRI expression by airway epithelium, peri-airway and interstitial infiltrating mononuclear cells, stromal cells, and alveolar macrophages.  No correlation between TGF-β or procollagen and AR or LB. Increased BALF TGF-β in transient or severe PGD2-3 vs PGD0-1 (both p<.01). Increased BALF procollagen in transient or severe PGD2-3 vs PGD0-1 (both p<.01). Association between transient or severe PGD2-3 and BOS, increased with PGD severity. |
| Devouassoux *et al*, 2001^14^ | Retrospective | 8 LTR (4 developed BOS)  9 non-LTx controls | TBB (first year post-LTx) | CD45, CD20, CD5, CD4, CD8, CD25, CD69, CD1  HLA class I and II, Ki-67, ELAM, LECAM, VCAM, ICAM, PECAM, VLA-4, LFA-1, Mac-1 | Increased leukocyte (CD45+) infiltration in grafts with future BOS (p=.003), CD20+ B-cells in LTR vs controls (p=.005), no difference in CD5+, CD4+, CD8+. More CD25+ and CD69+ cells in future BOS vs stable LTR (both p<.05).  Increased HLA class I expression in future BOS and stable LTR vs controls, decreased expression on alveolar macrophages in stable LTR (all p<.05). Increased Ki-67+ cells on capillary endothelium, alveolar and bronchial epithelium in grafts, and bronchial epithelium in future BOS vs stable LTR (all p<.05). Increased ELAM-1, VCAM-1, ICAM-1, VLA-4, LFA-1, Mac-1 expression on grafts, and VLA-4 in future BOS vs stable LTR (all p<.05). |
| Devouassoux *et al*, 2002^15^ | Retrospective  Longitudinal | 22 BOS  22 stable LTR | BALF | Differential cell count | Increased total cell count, neutrophils (%), eosinophils (%) and decreased macrophages (%) in BOS vs stable LTR (all p<.05). No difference in total cell count and neutrophils (%) in BOS 1 vs stable LTR, increased total cell count and neutrophils (%) in BOS 2 and 3 (all p<.05).  BOS < 12 mos. post-LTx was associated with rapid increase of neutrophils (1-3 mos. p<.01, 3-6 mos. p<.05), delayed increase of neutrophils if BOS > 12 mos. post-LTx (6-9 mos. p<.05, 9-12 mos. p<.01), > 9 mos. no difference between early and late BOS.  BOS 1: low neutrophilia, not influenced by BOS 1. BOS 2: low neutrophilia 3 mos. before diagnosis, increase at onset (p<.01), and peak 6 mos. later (p<.01). BOS 3: neutrophilia preceded diagnosis by 6 mos. (p<.01), peaked 9 mos. later (p<.01). |
| Elssner *et al*, 2000^16^ | Prospective | 10 BOS  9 stable LTR | BALF  BB | BALF: differential cell count, IL-8, IL-10, TGF-β, TNF-α  BB: IL-8, IL-10, TGB-β, TNF-α | Increased BALF neutrophils (#/%) and decreased macrophages (%) in BOS vs stable LTR (all p<.05).  Increased BALF IL-8 and TGF-β in BOS vs stable LTR (p<.005).  Higher IL-8 expression on bronchial epithelial cells (p<.05), not on BALF cells. No increased expression of TGF-β on BALF cells or bronchial epithelial cells in BOS vs stable LTR. No difference in IL-10 or TNF-α. |
| Fildes *et al*, 2008^17^ | Prospective  Cross-sectional | 9 BOS  21 stable TLR | TBB | NK cells (CD16)  Blood analyses (n=41 LTR) | Increased lung tissue NK cells in BOS vs stable LTR (p=.001).  Decreased but activated blood NK cells in BOS vs stable LTR. |
| Fisichella *et al*, 2013^18^ | Prospective  Longitudinal | 105 LTR (29 developed BOS) | BALF | Differential cell count, 34 cytokines, chemokines, and growth factors  Analyses in AR and aspiration | Increased lymphocytes (%) and neutrophils (%) and reduced macrophages (%) in BOS vs stable LTR (all p<.05).  Increased IL-1β, IL-8, CCL5, CXCL10, and decreased IL-9, IL-12 (p=.053), and CCL2 in BOS vs stable LTR (all p<.05).  Neutrophils (%) strongly correlated with time after LTx in BOS (p<.05).  Increased IL-15, IL-17, and TNF-α 6-12 mos. post-LTx predictive of early-onset BOS.  No difference in IL-1RA, IL-4, IL-5, IL-6, IL-7, IL-13, IL-15, IL-17, IFN-γ, TGF-β, TNF-α, CCL3, CCL4, and CCL11. |
| Gregson *et al*, 2010^19^ | Prospective | 47 LTR (13 developed BOS) | BALF  TBB | BALF: Tregs (CD3+CD4+CD25hi-FoxP3+), CCR4, CCR7, CD103, CD45RA subsets  TBB: CC21 expression | Tregs were essentially all CD45RA-, CCR4+, and CD103-.  No difference in total Treg frequency nor CCR4+ nor CD103- subsets in future BOS vs stable LTR.  Increased CCR7+ Tregs correlated with reduced risk of future BOS (p=.04).  CCR7 ligand CCL21 correlated with CCR7+ Treg frequency and inversely with BOS (both p<.05).  CCL21 protein is predominately expressed on bronchiolar epithelial cells and alveolar macrophages. |
| Hardison *et al*, 2009^20^ | Retrospective  Longitudinal | 7 BOS  8 AR  7 stable LTR | BALF | IL-8, MMP-8 and -9 activity and concentration  MPO, proline-glycine-proline (PGP), prolyl endopeptidase (PE), in vitro analyses | Increased IL-8 in BOS vs pre-BOS (p<.05).  Increased MMP-8 and MMP-9 concentration and activity in BOS vs stable LTR, pre-BOS, and AR (all p<.05).  Increased MPO in BOS vs stable LTR and pre-BOS (both p<.05). Increased PGP in BOS vs stable LTR, pre-BOS and AR (all p<.05). Increased PE detection and activity in BOS vs stable LTR, pre-BOS, and AR (all p<.01).  Correlation between MMP-9 activity and PGP levels (p<.05), PGP and PE (p<.01) and PGP and FVC (p<.05). |
| Hayes *et al*, 2020^21^ | Retrospective  Longitudinal | 16 CF LTR (10 developed BOS) | BALF | CD3, CD4, CD8 | Increased CD8+ and decreased CD4+ T-cells in BOS vs pre-BOS and vs stable LTR (all p<.05). No change in T-cell profile prior to BOS onset. No change over time in CD4+ or CD8+ in stable LTR. |
| Heigl *et al*, 2021^22^ | Retrospective  Cross-sectional | 14 BOS  16 RAS  13 stable LTR | BALF | C4d (ELISA)  AR and HLA-Ab analyses | Increased C4d in RAS vs stable LTR and BOS (both p<.01), not in BOS vs stable LTR.  Increased C4d in ACR, LB, AMR, and infection vs stable LTR (all p<.01).  Increased C4d in C4d (ICH)-/HLA-Ab-, C4d-/HLA-Ab+, C4d+/HLA-Ab+ vs stable LTR (all p<.05). Correlation between C4d and CRP (p<.0001). |
| Heijink *et al*, 2017^23^ | Retrospective  Cross-sectional | 20 BOS stage III  20 stable LTR | BALF (taken in stage I) | Differential cell count, MMP-1, -2, -3, -7, -8, -9, -12, -13, and TIMP-1, -2, -3, -4 concentration and activity | Increased neutrophils (%), lymphocytes (%), and IL-8 in BOS vs stable LTR (all p<.05).  Increased MMP-2, ‐3, ‐7, ‐8, and ‐9 levels in BOS vs stable LTR (all p<.05). Activity of MMP-7, but none of the other MMPs, was detected in stable LTR. No active MMPs in BOS. Increased TIMP-1 and -2 in BOS vs stable LTR (both p<.01). Increased TIMP-1-bound MMP-7, ‐8, and ‐9 and TIMP-2-bound MMP-8 and ‐9 levels in BOS vs stable LTR (all p<.0005).  MMP-3, -7, -8, -9 levels correlated with BALF neutrophil numbers in BOS. |
| Hodge G *et al*, 2009^24^ | Retrospective  Longitudinal | 12 BOS  35 stable LTR  18 healthy controls | BALF  BB | BALF: differential cell count, CD3, CD4, CD8, CD45/CD14  BB: CD4, CD8, CD3, CD45/CD14  In vitro T-cell cytokine production IL-2, IL-4, IFN-γ, TNF-α, blood analyses | No difference in BALF leukocytes (#), macrophages (#), T-cells (#). Increased CD8+ and decreased CD4+ in BOS and stable LTR vs controls (all p<.05).  No difference in BB T cell count (#/%), CD4+ or CD8+ cells.  More BALF CD8+ T-cells producing IFN-γ, IL-2, TNF-α in BOS and stable LTR vs controls, more CD4+ T-cells producing IFN-γ in BOS vs controls (all p<.05). No difference in BB cytokine production.  No difference in blood leukocytes (#), lymphocytes (#), T-cells (#). Increased blood CD8+ and decreased CD4+ in BOS and stable LTR vs controls. More blood CD4+ T-cells producing IL-2 in BOS and stable LTR vs controls, less blood CD4+ T-cells producing TGF-β in BOS and stable LTR vs controls. Less blood CD4+ and CD8+ T-cells producing TGF-β in BOS vs stable LTR. (all p<.05) |
| Hodge G *et al*, 2017^25^ | Retrospective  Cross-sectional | 8 BOS  18 stable LTR  10 healthy controls | BALF  BB | BALF/BB: CD3, CD4, CD8, NK T-like cells, NK cells (CD56)  Expression of granzyme B, perforin, in CD8+, CD4+, NK T-like and NK cells, and TNF-α, TFN-γ in CD8+, CD4+ and NK T-like cells, glucocorticoid receptor (GCR) expression, blood analyses | Decreased BALF T-cells and CD4+ T-cells, increased CD8+ T-cells, NK T-like, and NK cells (%) in BOS vs controls. Increased CD8+ T-cells in BOS vs stable LTR.  Increased large airway CD8+ T-cells, decreased CD4+ T-cells in BOS vs stable LTR.  Increased small airway CD8+ T-cells, NK T-like, NK cells, and decreased CD3+, CD4+ T-cells in BOS vs stable LTR and controls. (all p<.05)  More BALF CD8+ T-cells producing IFN-γ and TNF-α in BOS vs stable LTR and controls, and stable LTR vs controls. More CD8+ and CD4+ T-cells expressing granzyme B and perforin.  More large airway CD8+ T-cells and NK-cells producing IFN-γ and TNF-α in BOS vs stable LTR and controls. No difference in expression of granzyme B or perforin in T-cells, NK T-like, or NK cells, or GCR in CD8+ T-cells and NK cells.  More small airway CD8+ T-cells, NK T-like, and NK cells producing IFN-γ, TNF-α, granzyme B, perforin, and CD4+ T-cells granzyme B, perforin in BOS vs stable LTR and controls. And CD8+ IFN-γ, TNF-α, granzyme B in stable LTR vs controls. Less CD8+GCR+ T-cells in BOS vs stable LTR and controls and NKT-like GCR+ cells in BOS vs stable LTR. GCR expression by small airway CD8+ T-cells correlated with FEV1.  Decreased blood T-cell count, CD4+ T-cells and increased CD8+ T-cells, NK T-cell like (%) in BOS vs stable LTR and controls. Increased NK cells (%) in BOS and controls vs stable LTR. More blood CD8+ T-cells expressing IFN-γ, TNF-α, granzyme B, perforin, and less CD8+GCR+ T-cells in BOS vs stable LTR. More NK-cells expressing granzyme B, perforin in BOS vs stable LTR. More NK T-like cells expressing granzyme B, perforin in BOS and stable LTR vs controls. Less NK T-like GCR+ and NK GCR+ cells in BOS vs stable LTR and controls. |
| Hodge G *et al*, 2018^26^ | Retrospective  Cross-sectional | 12 BOS  18 stable LTR  13 healthy controls | BALF  BB | BALF/BB: CD3, CD4, CD8, NK T-like cells, NK cells (CD56)  Expression of TNF-α, IFN-γ, and HDA2c by T and NK T-like cells, blood analyses | Increased BALF CD8+ T-cells, NK T-like, NK cells, and decreased CD3+ and CD4+ (%) T-cells in BOS vs controls. Increased BALF CD8+ T-cells (%) in BOS vs stable LTR.  Increased large airway CD8+ and decreased CD4+ T-cells in BOS vs stable LTR. No changes in NK T-like and NK cells.  Increased small airway CD8+ T-cells, NK T-like, NK cells, and decreased CD3+, CD4+ T-cells in BOS vs stable LTR and controls. (all p<.05)  More BALF CD8+ and CD4+ producing IFN-γ and TNF-α in BOS vs stable LTR and controls, and CD8+ producing IFN-γ and TNF-α in stable LTR vs controls.  More large airway CD8+ T-cells and NK-cells producing IFN-γ and TNF-α in BOS vs stable LTR and controls. Less CD8+ T-cells and NK-cells expressing HDAC2 in BOS.  More small airway CD8+ T-cells producing IFN-γ and TNF-α in BOS vs stable LTR and controls and stable LTR vs controls. More NK T-like and NK cells producing IFN-γ and TNF-α in BOS vs stable LTR and controls. Less CD8+ T-cells and NK T-like cells expressing HDAC2 in BOS. HDA2c expression by small airway CD8+ T cells correlated with FEV1.  Increased blood CD8+ T-cells, NK T-like cells and decreased T-cells, CD4+ T-cells (%) in BOS vs stable LTR and controls. Increased NK cells (%) in BOS and controls vs stable LTR. More CD8+ T-cells expressing IFN-γ and TNF-α, loss of HDA2c expression by CD8+ T-cells, NK T-like and NK cells in BOS. |
| Hodge G *et al*, 2021^27^ | Retrospective  Cross-sectional | 10 BOS  11 stable LTR  10 healthy controls | BALF  BB | BALF/BB: CD3, CD4, CD8, NK T-like cells, NK cells (CD56)  Granzyme B, IFN-γ, TNF-α expression, blood analyses. | Decreased T-cells, increased NK T-like cells, CD8+ T-cells and NK T-like cells, CD28null CD8+ T-cells and NK T-like cells in BOS vs stable LTR and controls in BALF, large and small airway brushings, and blood (all p<.05).  Increased BALF granzyme B+ CD28null CD8+ T- and NKT-like cells in BOS vs stable LTR and controls, and stable LTR vs controls. Increased IFN-γ+ and TNF-α+ CD28null CD4+ and CD8+ T- and NK T-like cells in BOS vs stable LTR and controls.  Increased large airway granzyme B+ CD28null CD4+ and CD8+ T- and NK T-like cells in BOS vs stable LTR and controls, and stable LTR vs controls. Increased IFN-γ+ and TNF-α+ CD28null CD4+ and CD8+ T- and NK T-like cells in BOS vs stable LTR and controls, and TNF-α+ CD28null CD8+ T- and NK T-like cells in stable LTR vs controls.  Increased small airway granzyme B+ CD28null CD4+ and CD8+ T- and NK T-like cells in BOS vs stable LTR and controls, and stable LTR vs controls. Increased IFN-γ+ and TNF-α+ CD28null CD4+ and CD8+ T- and NK T-like cells in BOS vs stable LTR and controls, and stable LTR vs controls. Loss of CD28 expression by CD8+ T-cells was associated with FEV1.  Increased blood granzyme B+ CD28null CD4+ and CD8+ T- and NK T-like cells in BOS vs stable LTR and controls, increased granzyme B+ CD28null CD8+ T- and NK T-like cells and CD28null CD4+ NK T-like cells in stable patients vs controls. Increased IFN-γ+ and TNF-α+ CD28null CD8+ T- and NK T-like cells in BOS vs stable LTR and controls. |
| Hodge S *et al*, 2009^28^ | Prospective  Longitudinal | 6 BOS  16 stable LTR | BALF  BB | BALF: TGF-β1  BALF hepatocyte growth factor (HGF)  BB alpha smooth muscle actin (α-SMA), S100A4, extra-domain-A fibronectin (ED-A FN), HLA-DR | No difference in BALF TGF-β1 in BOS vs stable LTR.  Longitudinal increase of TGF-β1 in BOS vs pre-BOS (n=1).  Increased BALF HGF in BOS vs stable LTR (p<.05).  Increased bronchial epithelial cell expression of α-SMA, S100A4, ED-A FN, and HLA-DR in BOS vs stable LTR (all p<.05). Longitudinal increase of HGF, α-SMA, S100A4, and ED-A FN in BOS vs pre-BOS (n=1 LTR). |
| Hodge S *et al*, 2011^29^ | Retrospective  Cross-sectional | 25 BOS  34 infection  16 stable LTR  14 healthy controls | BALF | MBL, MBL-mediated C4d deposition Blood analyses, efferocytosis of apoptotic bronchial epithelial cells | Reduced MBL in BOS vs stable LTR and controls (both p<.05).  Reduced efferocytosis by alveolar macrophages from BOS vs stable LTR and controls (both p<.05). Increased plasma MBL and MBL-mediated C4d deposition in infected LTR vs stable LTR (both p<.05). Significant correlation between MBL and MBL-mediated complement deposition (p<.001), no correlation between blood and BALF MBL. |
| Hubner *et al*, 2005^30^ | Retrospective  Longitudinal | 8 BOS  12 stable LTR | BALF | Differential cell count, MMP-9, TIMP-1 concentration and activity | Increased neutrophils (%) and decreased macrophages (%) in BOS vs stable LTR and vs pre-BOS (both p<.05).  Increased MMP-9 and decreased TIMP-1 concentrations, and increased MMP-9/TIMP-1 ratio in BOS vs stable LTR (all p<.05). Increased MMP-9/TIMP-1 ratio in BOS vs pre-BOS (p<.05). Increased MMP-9 activity in BOS vs stable LTR, neutrophils were the main source.  MMP-9 correlated with neutrophil numbers and negatively with lymphocytes (both p<.02). MMP-9/TIMP-1 ratio correlated negatively with FEV1 (p=.003). |
| Ionescu *et al*, 2005^31^ | Retrospective  Longitudinal | 32 LTR (16 HLA-Ab+, 16 HLA-Ab-)  18 LTR with PGD or CMV pneumonitis | TBB | C4d deposition (IHC) | All subendothelial C4d+ HLA-Ab+ LTR developed BOS and/or graft loss.  More C4d deposition in HLA-Ab+ LTR vs HLA-Ab- (p<.05), all C4d+ cases were in LTR with DSA, no significant C4d deposition in PGD or CMV pneumonitis. |
| Kaes *et al*, 2020^32^ | Retrospective | 376 LTR | BALF | Differential cell count  Blood analyses | High BALF eosinophilia (≥ 2%) correlated with CLAD (p=.001) and CLAD-free survival (p=.003). Decreased CLAD-free survival in LTR with high blood and high BALF eosinophils, high blood and low BALF eosinophils, and low blood and high BALF eosinophils vs low blood and low BALF eosinophils (all p<.05); worst outcomes in LTR with high blood and high BALF eosinophils (p<.0001).  High blood eosinophils (≥ 8%) was associated with worse graft and CLAD-free survival (both p<.05). Within the high blood eosinophil group, 23.5% had RAS vs 3% in the low eosinophil group (p < 0.0001).  More episodes of LB and more severe LB, and more DSA in high vs low blood eosinophil group (all p<.05). |
| Keane *et al*, 2007^33^ | Prospective  Cross-sectional | 30 BOS  28 fBOS  10 treated BOS (tBOS)  47 stable LTR | BALF | IL-4, IL-13  Fibroblast proliferation, procollagen type I and III expression, in vitro and murine models | Increased IL-13 in BOS, fBOS, and tBOS vs stable LTR (all p<.05). No difference in IL-4.  Increased fibroblast proliferative response and procollagen type I and III expression in BOS, fBOS, and tBOS vs stable LTR (all p<.05).  Reduced fibroblast proliferation and procollagen type I and III expression in BOS, fBOS, and tBOS in the presence of anti-IL-13, no effect anti-IL-4 or anti-TGF-β. |
| Krustrup *et al*, 2015^34^ | Retrospective  Longitudinal | 58 LTR (28 developed BOS) | TBB | FoxP3+ Tregs | Highest number of FoxP3+ cells/mm² 2w post-LTx. No effect of FoxP3+ cells/mm² on BOS (p = 0.84), significant effect of A-score. The number of FoxP3+ cells/mm² after 2w did not predict the time interval to BOS (p=.65), also not as a time-dependent covariate (p=.77). |
| Laan *et al*, 2003^35^ | Prospective  Longitudinal | 7 BOS  7 stable LTR | BALF | Differential cell count, IL-16, IL-2R  AR analyses | No difference in IL-16 in BOS vs stable LTR at any time point (p=.6), no correlation with lymphocytes or IL-2R (p=.7).  Increased neutrophils (%) and decreased macrophages (%) in BOS vs stable LTR (both p<.05).  Lower IL-16 in AR vs stable LTR (p=.03), correlated negatively with IL-2R (p=.03), no correlation with lymphocytes. Increased lymphocytes (%) and decreased macrophages (%) in AR vs stable LTR (both p<.05). |
| Leonard *et al*, 2000^36^ | Prospective  Longitudinal | 8 BOS  14 stable LTR | TBB  EBB | Dendritic cell (DC) staining (CD1a, RFD1, MHC class II), suppressor macrophages (RFD1 and RFD7) | Increased CD1a and MHC class II DC in BOS vs stable LTR (both p<.05), increased DC using dendritic morphology and class II MHC expression vs CD1a expression (p<.000001). DC declined over time in BOS and stable LTR, no longer reaching statistical difference.  More MHC class II DC in EBB vs TBB (p<.003), no difference in CD1a DC.  No difference in DC CD80 or CD86 expression in BOS vs stable LTR. No difference in RFD1 staining vs CD1a staining.  No correlation between CD1a or MHC class II DC and AR or CMV pneumonitis. |
| Magro *et al*, 2003^37^ | Retrospective  Cross-sectional | 13 BOS  7 stable LTR  7 non-Tx controls | TBB | C1q, C4d, C5b-9, IgG, IgM, IgA (IF)  Pathology findings, serum anti-endothelial antibodies | Increased C1q, C3, C4d, C5b-9, and immunoglobulin deposition in the bronchial epithelium, chondrocytes, basement membrane zone of the bronchial epithelium, and bronchial wall microvasculature in BOS vs others. |
| Magro *et al*, 2003^38^ | Retrospective | 7 BOS | TBB | C4d, C3, C1q, C5b-9, IgG, IgM, IgA (IF)  AR analyses | Bronchial wall deposition of C1q, C4d, C5b-9, IgM, and IgA in BOS. Bronchial wall C1q deposition was the strongest predictor of BOS (p=.0038), C4d (p=.04), IgA (p=.04), and C5b-9 (p=.03).  C4d and c1q correlated with the degree of humoral rejection pathologically (both p<.01). No correlation with C3, C5b-9, and Ig. High and intermediate C4d levels correlated with a clinical diagnosis of AR (p<.0001). Absent or minimal C4d deposition correlated with a state of clinical wellbeing. No correlation between C4d deposition and presence of ACR. |
| Magro *et al*, 2006^39^ | Retrospective  Longitudinal | 24 LTR | TBB | C3d deposition (IF and IHC) | Intermediate and high levels of C3d correlated with BOS (p<.0001) and bronchial wall or septal fibrosis (p<.0016). All LTR with higher values of C3d within septae or bronchial wall eventually developed BOS.  Good correlation between C3d and C4d staining (p<.00001), no correlation between extent of C3d and ACR or AMR. IHC staining was superior to IIF. |
| Mamessier *et al*, 2007^40^ | Prospective | 13 BOS (7 stable and 13 evolving BOS samples)  7 AR  14 stable LTR | BALF | T-cell subtypes  Sputum and blood analyses | Increased CD4+CD25highCD69-Tregs (BALF, sputum, blood), Th1 (BALF, sputum IFN-γ+CD3+T cells), and Th2 (BALF, sputum IL13+CD3+, blood IL-4+CD3+cells), IL-13+ CD8+ T-cells (BALF, sputum), and IFN-γ+ CD8+ T-cells (BALF) in stable BOS vs stable LTR (all p<.05).  Increased Tregs (BALF, sputum, blood), Th1 (BALF, sputum), IFN-γ+ CD8+ T-cells (BALF) in evolving BOS vs stable LTR (all p<.05).  Higher Treg and Th2 activation and a lower Th1 activation in stable BOS vs evolving BOS (all p<.05).  Blood TGF-β was increased in AR and evolving BOS vs stable LTR (both p<.05). Increased blood IL-4 and TGF-β in evolving BOS vs stable LTR (both p<.01). Th1 activation was observed in AR. In AR, the proportion of Increased blood T cells expressing CD69 and CD103 in AR vs stable LTR, no difference in Tregs. |
| Meloni *et al*, 2004^41^ | Prospective  Longitudinal | 44 LTR (8 developed BOS) | BALF | Differential cell count, IL-8, IL-10, IL-12, IFN-γ, TGF-β, CCL2/MCP-1, CCL5/RANTES | Increased neutrophils (%), IL-8, CCL2, and decreased macrophages (%) and IL-12 in BOS vs stable LTR (all p<.05). Trend towards decreased TGF-β in BOS vs stable LTR (p=.06).  Lower levels of IL-12 were significantly predictive of BOS (p=.03). |
| Meloni *et al*, 2008^42^ | Retrospective  Longitudinal | 8 future BOS  8 stable LTR | BALF (at month 6) | CCL3/MIP1-α, CCL4/MIP1-β, CCL17/TARC, CCL19/MIP3-β, CCL20/MIP3-α, CCL22/MDC, CCL26/eotaxin  CCR4, CCR6, CCR7 on CD3+ and CD68+ cells | CCL19, CCL20, CCL22 levels at 6 mos. post-LTx predicted BOS onset (all p<.02), with a significantly different temporal trend in future BOS vs stable LTR.  No difference in CCR4, CCR6, CCR7 expression on CD3+ lymphocytes. Higher density of CCR6 in future BOS vs stable TLR (p=.02), no difference in CCR4.  Increased CCR6 and CCR4 expression on CD68+ cells in future BOS vs stable LTR (both p<0.02), trend CCR7 (p=.07). No difference in receptor density on CD68+ cells. |
| Neujahr *et al*, 2012^43^ | Prospective  Longitudinal | 40 LTR (15 developed BOS) | BALF during first year | IL1-RA, IL-13, IL-17, CCL2/MCP-1, CCL5/RANTES, CXCL9/MIG, CXCL10/IP-10 | Cumulative increased CXCL9 and CXCL10 were associated with BOS and graft failure (both p<.01), and preceded BOS onset by 3 and 9 mos. Sources of CXCL9 and CXCL10 were airway epithelium and alveolar macrophages.  No correlation between IL1RA, IL-13, IL-17, CCL2, CCL5 and BOS or graft failure. |
| Neurohr *et al*, 2009^44^ | Retrospective | 63 stable LTR (16 developed BOS) | BALF | Differential cell count, IL-8  Secretory leukocyte protease inhibitor (SLPI) | Increased total cell count and neutrophils (%) and decreased macrophages (%) in future BOS vs stable LTR (all p<.05).  Increased IL-8 in future BOS vs never BOS, and correlated with number of neutrophils (both p=.01).  BALF neutrophil percentage of ≥ 20% was a significant predictor for BOS ≥ 1 (p<.05).  Trend towards increased risk of death in future BOS (p=.056).  Reduced SLPI in future BOS vs stable LTR, correlated negatively with neutrophils (p=.01). |
| Ngo *et al*, 2019^45^ | Retrospective | 48 LTR | TBB | C4d deposition (IHC) | All C4d3+ LTR developed early persistent DSA, AMR, and CLAD (2 BOS, 1 RAS, 1 mixed).  Microvascular inflammation and acute lung injury were rare but more frequent in C4d1-3+, C4d+ was more frequent in infection. |
| Ramirez *et al*, 2008^46^ | Retrospective  Cross-sectional | 13 future BOS  21 stable LTR | BALF (last BALF before BOS onset) | TGF-β1, IL-1β, IL-2, IL-4, IL-5, IL-6, IL-8, IL-10, IL-12, IFN-γ, TNF-α, MMP-9 gelatinase activity  GM-CSF, in vitro fibronectin expression in murine fibroblasts | No difference in TGF-β1 level in future BOS vs stable LTR.  Higher MMP-9 activity in future BOS vs stable LTR (p<.005). Trend towards higher IL-8 in future BOS vs stable LTR (p=.08).  TGF-β1 correlated with fibronectin gene transcription (r=0.71). Higher fibronectin promoter activity in future BOS vs stable LTR (p=.026). |
| Reynaud-Gaubert *et al*, 2002^47^ | Retrospective | 26 LTR | BALF | Differential cell count, lymphocytes staining (CD3, CD4, CD8, CD37, CD57, HLA-DR) | Increased total cell count in BOS, AR, and infection vs stable LTR (p<.01). Increased neutrophils (%) in BOS and infection vs stable LTR (both p<.0001). Neutrophils outside the CI correlated with BOS (p<.01). Decreased macrophages (%) in BOS, AR, and infection vs stable LTR, and BOS and infection vs AR (all p<.01).  Increased CD4+ and decreased CD8+ cells in BOS and infection vs stable LTR (all p<.05).  Increased lymphocytes (%) in AR, decreased CD4+ T-cells in infection, increased HLA-DR+ lymphocytes (%) during infection and AR vs stable LTR, increased expression of NK-cell associated CD57 in infection vs others (all p<.05). |
| Reynaud-Gaubert *et al*, 2002^48^ | Prospective  Longitudinal | 21 LTR (8 developed BOS) | BALF | Differential cell count, IL-8, CCL2/MCP-1, CCL5/RANTES  Soluble intercellular adhesion molecule-1 (sICAM-1), vascular cell adhesion molecule-1 (VCAM-1) | Increased total cell count, neutrophils (#/%), and decreased macrophages (%) in BOS vs stable LTR (all p<.01).  Increased IL-8, CCL2, and CCL5 in BOS vs stable LTR (all p<.05). Increased neutrophils (#/%), IL-8, CCL2, and CCL5, and decreased macrophages (%) in future BOS vs stable LTR (all p<.05). Increased neutrophils (%) and IL-8 in post-BOS vs pre-BOS (both p=.02).  Neutrophils correlated with IL-8 and CCL2. Correlation between IL-8 and CCL2. Negative correlation between neutrophils and IL-8 and FEV1. (all p<.01)  No difference in sICAM-1 and VCAM-1 between groups. Neutrophils correlated negatively with sICAM-1. |
| Riise *et al*, 2010^49^ | Retrospective | 12 BOS  12 stable LTR | BALF | MMP-2 and MMP-9 concentration and gelatinase activity  Serine protease, neutrophil elastase, secretory leukocyte protease inhibitor | Increased net gelatinase activity in BOS vs stable LTR (p<.005). MMP-9 activity exceeded MMP-2 activity in BOS and stable LTR (both p<.01). Increased MMP-9 activity and concentration in BOS vs stable LTR (both p<.05), but not MMP-2.  Gelatinase activity correlated with MMP-9 concentration and neutrophils (%) (both p<.01).  Increased neutrophil elastase in BOS vs stable LTR (p<.01). Serine protease correlated with neutrophil elastase concentration and neutrophils (both p<.05). |
| Sacreas *et al*, 2019^50^ | Retrospective  Cross-sectional | 1. BALF: 23 BOS, 26 RAS, 20 stable LTR 2. Explant lungs: 19 BOS, 19 RAS, 14 non-LTx controls | BALF  Explant lungs | BALF: TGF-β1  Explant lungs: TGF-β1, CD4, CD8, CD20, CD68  Calretinin, serum mesothelin, explant lung CT and protein and mRNA analyses, in vitro analyses | Increased BALF TGF-β1 in RAS vs stable LTR (p = 0.02).  Worse post-diagnosis graft survival in RAS LTRs with high TGF-β1 levels vs those with low TGF-β1 levels (p = 0.033).  TGF-β1 was located in the (sub)pleural area in explant lungs. Low TGF-β1 expression: little and dispersed CD4+, CD20+, and CD68+ cells. Intermediate TGF-β1 expression: low number of CD4+, CD8+, CD20+, and CD68+ cells. High TGF-β1 expression: higher number of CD4+, CD8+, CD20+, and CD68+ cells.  Increased volume fraction of pleura in RAS explant lungs vs BOS and non-LTx controls, higher proportion of calretinin+ staining in RAS vs BOS and controls, decreased E-cadherin mRNA expression in RAS pleura vs controls, increased α-smooth muscle actin mRNA and protein expression in RAS pleura vs controls, increased blood mesothelin in RAS vs stable LTR (all p<.05). TGF-β1 stimulation of pleural mesothelial cells led to a phenotypical switch to mesenchymal cells, accompanied with an increased migratory capacity. IL-1α was able to accentuate TGF-β1‒induced mesothelial-to-mesenchymal transition. |
| Saito *et al*, 2013^51^ | Retrospective  Cross-sectional | 50 BOS  21 RAS  38 stable LTR | Donor lung biopsies | IL-1β, IL-6, IL-8, IL-10, IFN-γ, TNF-α mRNA | Increased IL-6 expression in pre-implanted lungs from future BOS patients vs RAS and stable LTR (both p<.03).  Increased IL-1β and IL-6 in future CLAD vs no-CLAD (both p<.05).  Association between high IL-6 and CLAD, BOS, and early BOS development (all p<.01). |
| Saito *et al*, 2018^52^ | Retrospective  Cross-sectional | 18 BOS  10 RAS  25 stable LTR | BALF | MMP-8  Neutrophil elastase, α-defensins, long pentraxin-3 (PTX3) | Upregulated MMP-8 in RAS (p<.001) and BOS (p=.002) vs stable LTR.  Upregulated neutrophil elastase, α-defensins, and PTX3 in RAS vs stable LTR (all p<.001), neutrophil elastase, α-defensins and PTX3 vs BOS (all p<.01), and neutrophil elastase in BOS vs stable LTR (p=.024). |
| Sato *et al*, 2009^53^ | Retrospective  Cross-sectional | 12 explant BOS lungs  1 surgical BOS biopsy  15 non-LTX controls | Explant lungs | CD3, CD20, CD45RO, CCR7, lymphocyte aggregations  Peripheral lymph node addressin (PNAd), high endothelial venules (HEV), Ki-67, animal analyses | More T- and B-cells in LB and active OB vs inactive OB and controls. T-cells in LB and active OB lesions were mainly CD45RO+ CCR7- effector memory T-cells.  Effector memory T-cell aggregates did not completely meet the anatomical criteria of secondary or tertiary lymphoid tissue, because they did not include segregated T-cell and B-cell zones or B-cell follicles positive for CD21+ follicular dendritic cells.  Large number of PNAd+ HEVs in the airways of BOS lungs vs controls (p<.01). HEVs existed in almost all of the LB and active OB lesions in the bronchiolar wall vs a small number of HEVs in inactive OB lesions. |
| Sato *et al*, 2011^54^ | Retrospective  Cross-sectional | 20 CLAD explant lungs  20 non-LTx controls  20 LTR (7 developed CLAD) | CLAD explant lungs  TBB | CXCL12, CXCL13, CCL21  Peripheral lymph node addressin (PNAd) | Increased lymphoid aggregates, CXCL12 in alveolar and airway epithelial cells, CCL21+ lymph vessels (all p<.01), and infiltration of DC-specific intercellular adhesion molecule-grabbing nonintegrin+ immature DCs (p=.056) in CLAD explant lungs vs controls.  Increased PNAd+ high endothelial venule like vessels in CLAD explant lungs vs controls, and in TBB of future CLAD vs stable LTR (all p≤0.001). |
| Scholma *et al*, 2000^55^ | Prospective  Cross-sectional | 60 LTR (19 developed BOS) | BALF on mean day 41 | Differential cell count, IL-6, IL-8, CCL2 | Total cell count (#), lymphocytes (#), eosinophils (#), IL-6, and IL-8 were higher in future BOS vs stable LTR in bronchial fraction (all p<.05).  Increased neutrophils (#), IL-6, and IL-8 in alveolar fraction in future BOS vs stable LTR (p<.05). No difference in CCL2.  Increased total cells, neutrophils, lymphocytes, eosinophils, IL-6, and IL-8 in bronchial fraction and total cells, neutrophils, IL-6, IL-8, and CCL2 in alveolar fraction correlated with increased BOS risk. |
| Shino *et al*, 2013^56^ | Retrospective  Longitudinal | 224 LTR | BALF | CXCL9/MIG, CXCL10/IP-10, CXCL11/ITAC  Pathologic findings of 441 LTR | Prolonged elevation of CXCR3 ligands (CXCL9, -10, -11) correlated with increased CLAD risk.  CXCL9, CXCL10, and CXCR3 were expressed by epithelial cells, mononuclear cells, and alveolar macrophages. CXCL11 was mainly expressed by vascular endothelial cells.  Increased CXCL9 and CXCL10 during DAD, AR, and LB, and CXCL11 during DAD. Episode of DAD correlated strongly with increased risk of CLAD, especially RAS, and allograft failure. AR and LB correlated with increased risk of CLAD, AR correlated with BOS and RAS. OP correlated with allograft failure. (all p<.05) |
| Sinclair *et al*, 2021^57^ | Retrospective  Cross-sectional | 7 CLAD  7 early stable LTR (< 1y)  7 stable LTR (> 1y)  7 healthy controls | BALF | CCL2, TGF-β  Hepatocyte growth factor (HGF), epidermal growth factor (EGF), platelet derived growth factor BB (PDGF-BB), lysophosphatidic acid (LPA), autotaxin, mesenchymal stromal cells (MSc) migration, in vitro analyses | Increased CCL2 in early post-LTx, CLAD, and stable LTR vs controls (all p<.05). No difference in TGF-β in CLAD vs stable LTR.  CLAD BALF increased MSc migration (all p<.05), BALF from healthy controls and early post-LTx LTR (<1j) did not induce MSc migration.  Increased HGF in CLAD vs early post-LTx and controls. Increased autotaxin in early post-LTx, CLAD, and stable LTR vs controls. Autotaxin mRNA was increased in LTR who developed CLAD early post-LTx, autotaxin expression was inversely correlated with time to CLAD.  No difference in EGF and PDGF-BB. Increased LPA species 16:0 and 22:4 in LTR vs controls. LPA inhibition completely blocked the effect of CLAD BALF on chemotaxis. |
| Snell *et al*, 2007^58^ | Prospective  Longitudinal | 34 stable LTR | BALF  EBB | BALF: differential cell count, CD3, CD8, IL-8  EBB: CD3, CD4, CD8, CD45, IL-17 | No correlation between EBB IL-17+ cells and BALF IL-8, neutrophils (%), acute rejection, or BOS.  EBB IL-17 was elevated early and subsequently fell with time post-LTx.  EBB IL-17 correlated with EBB CD8+ cells, increased BALF lymphocytes, and correlated negatively with time post-LTx (all p<.05).  EBB IL-17 increased in CMV mismatch and clinical infection (all p<.05). |
| Suwara *et al*, 2014^59^ | Retrospective  Longitudinal | 9 RAS  13 BOS  10 LB/ARAD  10 persistent airway neutrophilia (PAN)  13 stable LTR | BALF | Differential cell count, IL-1α, IL-1β, IL-6, IL-8, TNF-α  Plasma CRP, in vitro viability of bronchial epithelial cells | Increased leukocytes (#) in ARAD, PAN, pre-BOS, and BOS vs controls. Increased neutrophils (%) in ARAD, PAN, BOS, and RAS. (all p<.01) No difference in neutrophils in pre-BOS or pre-RAS vs stable LTR. Increased eosinophils (%) in RAS vs control (p=.01). Decreased macrophages (%) in ARD, PAN, and RAS vs controls (all p<.05).  Increased IL-1α and IL-1β in ARAD and PAN vs controls, increased IL-6 in PAN and RAS vs controls, increased IL-8 in ARAD, PAN, and BOS vs controls, increased TNF-α in PAN vs controls (all p<.05).  Increased IL-1α in BOS vs pre-BOS (p=.02).  Increased plasma CRP in PAN and RAS vs controls (both p<.01). Decreased epithelial cell viability after exposure to BALF in PAN (p<.01). |
| Suzuki *et al*, 2013^60^ | Retrospective  Cross-sectional | BALF: 6 BOS, 10 non-LTx controls  Tissue: 4 BOS,  4 non-LTx controls | BALF  Explant lungs | BALF: C3a  Tissue: CD55, CD46  In vitro, murine analyses | Upregulation of BALF C3a in BOS vs controls (p<.05).  Downregulation of tissue complement-regulatory protein (CD55, CD46) in BOS vs controls (both p<.05). |
| Vanaudenaerde *et al*, 2008^61^ | Retrospective  Cross-sectional | 36 BOS  11 infection  43 AR  42 stable LTR | BALF | IL-1β, IL-2, IL-6, IL-8, IL-17, IL-23, TGF-β | Increased neutrophils (#/%) in BOS, infection, and AR vs stable LTR (all p<.01). Increased lymphocytes (#) in BOS, infection, and AR vs stable LTR (all p<.05). Decreased macrophages (%) in BOS and infection vs stable LTR (both p<.001).  Increased IL-1β, IL-6, IL-17 mRNA, IL-23 mRNA, TGF-β mRNA, and decreased IL-2 in BOS vs stable LTR (all p<.05).  Increased IL-8 protein in BOS, infection, and AR vs stable LTR, increased IL-8 mRNA in BOS vs stable LTR (all p<.05).  TGF-β protein levels did not significantly differ.  Increased IL-1β, IL-6, IL-17, IL-23, TGF-β mRNA in infection vs stable LTR (all p<.05). Increased IL-6 and decreased IL-2 in AR vs stable LTR.  Increased total cell count (#) in infection and AR vs stable LTR (both p<.001). Increased macrophages (#) in infection and AR vs stable LTR (both p<.01). Increased eosinophils (#/%) in infection vs stable LTR (p<.05). |
| Vandermeulen *et al*, 2015^62^ | Retrospective  Cross-sectional | 72 stable LTR with high BALF neutrophilia (≥ 15%)  37 stable LTR with low BALF neutrophilia | BALF | Differential cell count, 33 cytokines, chemokines, and growth factors | Increased total cell count, neutrophils (#/%), eosinophils (#), lymphocytes (#), IL-1β, IL-1RA, IL-4, IL-6, IL-8, IL-9, IL-10, TNF-α, CCL2, CCL3, CCL4, CCL5, CCL11, CXCL8, CXCL10, and decreased macrophages (%) in neutrophil-high vs neutrophil-low group (all p<.05).  Correlation between IL-8 and BALF neutrophilia. Correlation between IL-1β and IL-8, markers of eosinophils (IL-4, CCL11) and markers of macrophages (CCL2, CCL3, CCL4) (all p<.05), correlating trend IL-1β and CLAD-free survival (p=.084).  Increased CLAD incidence, lower CLAD-free, and overall survival in neutrophil-high vs neutrophil-low group (all p<.05).  No difference in IL-2, IL-7, IL-12, IL-13, IL-15, IL-17, IFN-γ, CXCL5, CXCL6, CCL18, CCL22.  Increased FGF-β, G-CSF, PDGF, VEGF (%) in neutrophil-high vs neutrophil-low group (all p<.05). No difference in GM-CSF. |
| Vandermeulen *et al*, 2016^63^ | Retrospective  Cross-sectional | 15 BOS  16 RAS  14 stable LTR | BALF | Differential cell count, C4d, C1q, IgA, IgE, IgG1-4, IgG, IgM, proMMP-2, proMMP-9, MMP9 | Increased total cell count in BOS vs stable LTR, increased neutrophils (#/%), lymphocytes (#), and decreased macrophages (%) in BOS and RAS vs stable LTR, increased macrophages (#) in BOS vs stable LTR and RAS, increased eosinophils (#/%) in RAS vs stable LTR (all p<.05).  Increased IgG, IgG1-4, IgM in RAS vs stable LTR and BOS. Increased IgA and IgE in RAS vs stable LTR, and increased total IgG and IgE in BOS vs stable LTR (all p<.05).  Increased IgG, IgG1, IgG3, IgG4, IgM correlated with worse survival (all p<.05).  Increased C4d and C1q in RAS vs BOS and stable LTR, and correlated with mortality and IgG1-4, IgG, IgE, IgA (all p<.05).  Increased proMMP-9 and MMP-9 levels and activated MMP-9 in RAS and BOS vs stable LTR, and increased MMP-9 induced gelatin degradation in BOS vs stable LTR. Increased proMMP-2 in RAS vs stable LTR. (all p<.01)  Increased blood DSA in RAS vs BOS and stable LTR (p=.017). |
| Vandermeulen *et al*, 2017^64^ | Retrospective  Cross-sectional | 19 BOS  18 RAS  21 non-Tx controls | Explant lungs (BOS/RAS)  Biopsies (controls) | Neutrophils (MPO), eosinophils (EG-2), macrophages (CD68), mast cells (tryptase), dendritic cells (CD1a, CD207), B-cells (CD20), cytotoxic T-cells (CD8), T-helper cells (CD4), lymphoid follicles  DSA | Increased number neutrophils, eosinophils, macrophages, mast cells, B-cells, and cytotoxic T-cells in RAS vs controls (all p<.05). Increased B-cells and cytotoxic T-cells in BOS vs controls (both p<.05). Lymphoid follicles in RAS vs BOS and controls, predominantly localized around the blood  vessels and in the parenchyma (all p<.05).  Myeloid cell types were more prevalent around the airways vs parenchyma or around blood vessels. Increased neutrophils in airway component in RAS and BOS vs controls. Eosinophils and mast cells in RAS were primarily located in the parenchyma and around blood vessels. Macrophages were more abundant in RAS vs controls and BOS in every compartment. More CD1a dendritic cells in the parenchyma in RAS vs BOS and controls. Increased resident mucosal (langerin positive) DC in the parenchyma in RAS vs controls and decreased around the airways in RAS vs controls. (all p<.05)  DSA were more prevalent in RAS vs BOS (p=.04). |
| Verleden *et al*, 2011^65^ | Retrospective  Cross-sectional | 9 fBOS  9 NRAD  10 stable LTR | BALF | Differential cell count, 32 cytokines, chemokines, growth factors | Increased neutrophils (%) and eosinophils (%) and decreased macrophages (%) in CLAD vs stable LTR. Increased total cell count, neutrophils (%) and decreased macrophages (%) in NRAD vs stable LTR (all p<.05).  Upregulated IL-1β, IL-8, MMP-8, MMP-9, MMP-8/TIMP-1, MMP-9/TIMP-1 in CLAD/NRAD vs stable LTR (all p<.05). No difference in fBOS vs stable LTR.  Upregulated IL-1β, IL-8, CCL2, CCL5, TIMP-1, MMP-8, MMP-9 in NRAD vs fBOS (all p<.05).  IL-1β, IL-8, CCL2, CCL5, TIMP-1, MMP-8, and MMP-9 correlated with BALF neutrophils (%).  No differences in TNF-α and TGF-β.  Upregulated HGF, MPO, and downregulated RAGE, SP-C, and PDFG-AA in NRAD vs stable LTR. Upregulated HGF, MPO, bile acids, and downregulated PDGF-AA in NRAD vs fBOS. FGFb, PLGF, HGF MPO, RAGE, SP-C, and bile acids correlated with BALF neutrophils (%). |
| Verleden *et al*, 2014^66^ | Retrospective  Longitudinal | 66 LTR with eosinophilia (≥ 2%)  253 LTR without eosinophilia | BALF | Differential cell count  Blood analyses | Increased BALF eosinophilia (≥ 2%) correlated with worse CLAD-free and overall survival vs controls (both p<.01), and predisposed to BOS and especially RAS (p<.0001).  Higher CLAD and mortality risk if multiple BALF eosinophilia vs once (both p<.01).  Correlation between BALF (%) and blood eosinophilia (%) (p<.0001), higher blood eosinophils in CLAD LTR in eosinophil group vs those who did not develop CLAD (p=.07). Increased CRP in eosinophil group vs controls (p<.0001). |
| Verleden *et al*, 2015^67^ | Retrospective  Cross-sectional | 20 BOS  17 neutrophilic BOS  20 RAS  20 stable LTR | BALF | Differential cell count, 34 cytokines, chemokines, growth factors | Increased total cell count and neutrophils (#/%) and decreased macrophages (%) in neutrophilic BOS and RAS vs non-neutrophilic BOS and stable LTR, and higher eosinophils (%) in RAS vs BOS and stable LTR (all p<.05).  Upregulated IL-1β, IL-1Rα, IL-6, IL-8/CXCL8, CCL2, CCL3, CCL4, CXCL10 and decreased VEGF in RAS vs stable LTR (all p<.05).  Increased IL-1Rα, IL-6, IL-8, CCL3, CCL4 in RAS vs non-neutrophilic BOS and IL-6, CCL18, and decreased VEGF vs neutrophilic BOS (all p<.05).  Upregulated IL-1β, IL-1Rα, IL-4, IL-7, IL-8/CXCL8, CCL3, CCL4, CCL7, and decreased CCL18 in neutrophilic BOS vs stable LTR (all p<.05) and upregulated IL-1β, IL-7, IL-8, CCL3, CCL7 and decreased CCL18 vs non-neutrophilic BOS.  No difference between non-neutrophilic BOS and stable LTR.  IL-6, CXCL10, CXCL11 were associated with survival after diagnosis in RAS (all p<.05).  No difference in IFN-γ, TNF-α, IL-2, IL-5, IL-9, IL-10, IL-13, IL-17, CCL5, CXCL5, CXCL6, CXCL9, and CXCL11. |
| Verleden *et al*, 2016^68^ | Retrospective  Cross-sectional | 33 RAS | BALF | Differential cell count  Radiology, pathology and pulmonary function test findings, blood eosinophilia (n=53) | Increased neutrophils and eosinophils (%) and decreased macrophages (%) correlated with worse graft survival (all p<.05). Strong association between increased eosinophils (≥ 2%) and survival after diagnosis (p=.0002), and neutrophils (> 10%) and survival (p=.019).  BALF eosinophilia correlated with blood eosinophilia (#/%). Increased blood eosinophilia (#) and lower lobe or diffuse infiltrates correlated with worse graft survival. Blood eosinophil count > 240 x 10^6^/L correlated with worse outcome (p=.0015). |
| Verleden *et al*, 2018^69^ | Retrospective  Cross-sectional | 336 LTR | BALF < 24-48h post-LTx | Differential cell count, IL-6, IL-8 | High IL-6 < 24h post-LTx was associated with better CLAD-free and graft survival (both p<.05).  Weak correlation between neutrophilia (%) and IL-6 and inverse correlation with macrophages (both p<.05).  IL-8 correlated with IL-6 (p<.0001).  Increased IL-6 < 24h post-LTx was associated with longer ICU and hospital stay and increased PGD3 prevalence (all p<.01).  Increased IL-8 < 24h post-LTx correlated with PGD3 and ECMO use, higher donor paO2, younger donor age, but not with other short-or long-term outcome (p<.01). |
| Vos *et al*, 2009^70^ | Retrospective  Cross-sectional | 121 LTR (54 developed BOS) | BALF | Differential cell count, IL-6, IL-8  BALF and plasma CRP | Increased neutrophils (#/%) in BOS vs stable LTR (p<.03). Trend for increased total cell count in BOS vs stable LTR (p=.053). No difference in IL-6 or IL-8.  BALF CRP at D90 was an independent predictor for graft failure (p=.004), trend for plasma CRP (p=.077). Increased BALF and plasma CRP in BOS vs stable LTR (p<.03 resp. p=.056). Increased plasma CRP, BALF CRP and neutrophils in AR vs without AR (all p≤.02). Increased BALF neutrophils in colonized vs non-colonized LTR (p=.047). |
| Ward *et al*, 2001^71^ | Retrospective  Cross-sectional | 5 BOS  19 stable LTR  18 healthy controls | BALF | Differential cell count, lymphocyte, alveolar macrophages (AM), and NK surface markers  (CD3, CD45, CD4, CD8, CD14, CD25, HLA-DR, CD56, CD16, CD11a, CD11b, CD11c, CD18) | Increased neutrophils (%) in stable LTR and BOS vs controls (p<.05) and trend in BOS vs stable LTR (p=.08).  Increased NK cells (CD56/CD16+), CD11b+ and CD11a+ CD3+ lymphocytes, CD8+ lymphocytes, and decreased CD4+ cells (%) in stable LTR and BOS vs controls (all p<.05). Increased CD11a CD3+ lymphocytes in stable LTR vs controls (p<.05). Decreased expression of AM surface markers CD11a, CD11b, CD11c, HLA-DR and CD14 in stable LTR and BOS vs controls (all p<.05).  Increased HLA-DR expression in CD8+ cells in stable LTR and BOS vs controls (all p<.05). |
| Westall *et al*, 2008^72^ | Retrospective  Cross-sectional | 33 LTR (9 developed early BOS, mean 79 weeks) | TBB first 3 months post-LTx | C3d and C4d deposition (IHC)  Pathologic findings, correlation AMR, PGD, respiratory infection, CMV pneumonitis | Early (< 3 mos. post-LTx) C3d/C4d deposition was not associated with ACR, lung function, BOS, or mortality.  9 LTR with early BOS all had significant intracapillary C3d/C4d deposition and features of AMR.  Good correlation between C3d and C4d staining (p<.05), no correlation between degree and extent of C3d/C4d and morphologic features of AMR, increased C3d/C4d deposition in severe PGD3 (p=.07/.01) and respiratory infection (p=.01/.02). No association between C3d/C4d deposition and CMV pneumonitis. |
| Yang *et al*, 2019^73^ | Retrospective  Cross-sectional | 20 BOS  20 RAS  20 stable LTR | BALF | Differential cell count, IL-6, IL-8, CXCL10/IP-10  Cell-free DNA (cfDNA) | Increased neutrophils (%) and decreased macrophages (%) in BOS and RAS vs stable LTR (all p=.01). No difference in eosinophils.  Higher IL-8 in BOS vs stable LTR (p=.0163), no differences in IL-6. Trend towards higher CXCL10 in RAS vs stable LTR (p=.08).  Higher cfDNA in BOS vs RAS and stable LTR (both p<.01).  Association between overall survival and cfDNA, CXCL10, and cfDNA-CXCL10 interaction (all p<.05). |
| Zheng *et al*, 2000^74^ | Retrospective  Cross-sectional | 10 BOS  19 stable LTR  20 healthy controls | BALF  TBB, EBB | BALF: differential cell count, IL-8  TBB/EBB: neutrophil elastase staining | Increased BALF neutrophilia (#/%) in BOS vs stable LTR and controls, and in stable LTR vs controls (all p<.01). Decreased alveolar macrophages (%) in BOS vs controls and stable LTR (both p<.01).  Increased BALF IL-8 in BOS vs stable LTR and controls, and stable LTR vs controls (all p<.01). BALF IL-8 strongly correlated with neutrophils (%) in BOS (p<.05), not in stable LTR.  Increased airway wall neutrophilia in BOS and stable LTR vs controls (both p<.05). No difference in neutrophils in lung parenchyma in BOS vs stable LTR. |
| Zheng *et al*, 2005^75^ | Prospective  Longitudinal | 29 LTR (23 developed BOS0p, 17 BOS ≥ 1)  6 healthy controls | BALF  EBB | BALF/EBB: differential cell count, CD3, CD4, CD8, HLA-DR | Increased BALF total cell count in LTR vs controls, no difference in lymphocytes (%) in LTR vs controls, decreased lymphocytes (%) in BOS vs pre-BOS (p=.057).  Decreased BALF CD3+ over time in BOS, and after BOS vs pre-BOS (all p<.05). Increased BALF CD8+ and decreased CD4+ early post-LTx vs controls (all p<.05). No difference in CD4 or CD8 in BOS vs pre-BOS.  Increased EBB CD3+ and CD8+ lymphocytes over time vs early post-LTx, and more pronounced in BOS (all p<.05). No difference after BOS vs pre-BOS, trend towards higher CD8+ infiltration in BOS vs stable LTR. |
| Zheng *et al*, 2006^76^ | Prospective  Longitudinal | 28 stable LTR (16 developed BOS)  15 healthy controls | BALF  EBB | BALF: differential cell count, IL-8  EBB: lymphocytes (CD3, CD4, CD8), macrophages (CD68), neutrophils (neutrophil elastase) | Increased BALF baseline total cell counts, neutrophils (%), and IL-8 in never BOS and future BOS vs controls (all p<.05), and persisted over time. No difference in BALF lymphocytes and macrophages (%).  EBB lymphocytes and macrophages (#) were similar to controls at baseline, but increased over time.  EBB neutrophils were similar to controls at baseline, but increased over time in BOS (p=.0004).  Increased EBB and BALF neutrophils and BALF IL-8 in post-BOS vs pre-BOS (all p<.01). BALF IL-8 correlated with BALF neutrophils (%) (p<.001) and EBB neutrophils (#) (p=.01).  Increased BALF neutrophils (%), but not EBB neutrophils (#), in case of bronchopulmonary infection in LTx patients who developed BOS (p=.002). In the presence of concomitant infections, BALF neutrophilia was more marked post-BOS vs pre-BOS (p=.01). |

**References**

1. Agostini C, Calabrese F, Rea F, et al. Cxcr3 and its ligand CXCL10 are expressed by inflammatory cells infiltrating lung allografts and mediate chemotaxis of T cells at sites of rejection. *Am J Pathol.* 2001;158(5):1703-1711.

2. Banerjee B, Ling KM, Sutanto EN, et al. The airway epithelium is a direct source of matrix degrading enzymes in bronchiolitis obliterans syndrome. *J Heart Lung Transplant.* 2011;30(10):1175-1185.

3. Banga A, Han Y, Wang X, Hsieh FH. Mast cell phenotypes in the allograft after lung transplantation. *Clin Transplant.* 2016;30(7):845-851.

4. Belperio JA, Keane MP, Burdick MD, et al. Critical role for the chemokine MCP-1/CCR2 in the pathogenesis of bronchiolitis obliterans syndrome. *J Clin Invest.* 2001;108(4):547-556.

5. Belperio JA, Keane MP, Burdick MD, et al. Critical role for CXCR3 chemokine biology in the pathogenesis of bronchiolitis obliterans syndrome. *J Immunol.* 2002;169(2):1037-1049.

6. Belperio JA, DiGiovine B, Keane MP, et al. Interleukin-1 receptor antagonist as a biomarker for bronchiolitis obliterans syndrome in lung transplant recipients. *Transplantation.* 2002;73(4):591-599.

7. Berastegui C, Gómez-Ollés S, Sánchez-Vidaurre S, et al. BALF cytokines in different phenotypes of chronic lung allograft dysfunction in lung transplant patients. *Clin Transplant.* 2017;31(3).

8. Bhorade SM, Chen H, Molinero L, et al. Decreased percentage of CD4+FoxP3+ cells in bronchoalveolar lavage from lung transplant recipients correlates with development of bronchiolitis obliterans syndrome. *Transplantation.* 2010;90(5):540-546.

9. Borthwick LA, Corris PA, Mahida R, et al. TNFα from classically activated macrophages accentuates epithelial to mesenchymal transition in obliterative bronchiolitis. *Am J Transplant.* 2013;13(3):621-633.

10. Budd SJ, Aris RM, Medaiyese AA, Tilley SL, Neuringer IP. Increased plasma mannose binding lectin levels are associated with bronchiolitis obliterans after lung transplantation. *Respir Res.* 2012;13(1):56.

11. Calabrese DR, Chong T, Wang A, et al. NKG2C Natural Killer Cells in Bronchoalveolar Lavage Are Associated With Cytomegalovirus Viremia and Poor Outcomes in Lung Allograft Recipients. *Transplantation.* 2019;103(3):493-501.

12. Carroll KE, Dean MM, Heatley SL, et al. High levels of mannose-binding lectin are associated with poor outcomes after lung transplantation. *Transplantation.* 2011;91(9):1044-1049.

13. DerHovanessian A, Weigt SS, Palchevskiy V, et al. The Role of TGF-β in the Association Between Primary Graft Dysfunction and Bronchiolitis Obliterans Syndrome. *Am J Transplant.* 2016;16(2):640-649.

14. Devouassoux G, Pison C, Drouet C, Pin I, Brambilla C, Brambilla E. Early lung leukocyte infiltration, HLA and adhesion molecule expression predict chronic rejection. *Transpl Immunol.* 2001;8(4):229-236.

15. Devouassoux G, Drouet C, Pin I, et al. Alveolar neutrophilia is a predictor for the bronchiolitis obliterans syndrome, and increases with degree of severity. *Transpl Immunol.* 2002;10(4):303-310.

16. Elssner A, Jaumann F, Dobmann S, et al. Elevated levels of interleukin-8 and transforming growth factor-beta in bronchoalveolar lavage fluid from patients with bronchiolitis obliterans syndrome: Proinflammatory role of bronchial epithelial cells. *Transplantation.* 2000;70(2):362-367.

17. Fildes JE, Yonan N, Tunstall K, et al. Natural killer cells in peripheral blood and lung tissue are associated with chronic rejection after lung transplantation. *J Heart Lung Transplant.* 2008;27(2):203-207.

18. Fisichella PM, Davis CS, Lowery E, Ramirez L, Gamelli RL, Kovacs EJ. Aspiration, localized pulmonary inflammation, and predictors of early-onset bronchiolitis obliterans syndrome after lung transplantation. *J Am Coll Surg.* 2013;217(1):90-100; discussion 100-101.

19. Gregson AL, Hoji A, Palchevskiy V, et al. Protection against bronchiolitis obliterans syndrome is associated with allograft CCR7+ CD45RA- T regulatory cells. *PLoS One.* 2010;5(6):e11354.

20. Hardison MT, Galin FS, Calderon CE, et al. The presence of a matrix-derived neutrophil chemoattractant in bronchiolitis obliterans syndrome after lung transplantation. *J Immunol.* 2009;182(7):4423-4431.

21. Hayes D, Jr., Harhay MO, Nicol KK, Liyanage NPM, Keller BC, Robinson RT. Lung T-Cell Profile Alterations are Associated with Bronchiolitis Obliterans Syndrome in Cystic Fibrosis Lung Transplant Recipients. *Lung.* 2020;198(1):157-161.

22. Heigl T, Saez-Gimenez B, Van Herck A, et al. Free Airway C4d after Lung Transplantation - A Quantitative Analysis of Bronchoalveolar Lavage Fluid. *Transpl Immunol.* 2021;64:101352.

23. Heijink IH, Rozeveld D, van der Heide S, et al. Metalloproteinase Profiling in Lung Transplant Recipients With Good Outcome and Bronchiolitis Obliterans Syndrome. *Transplantation.* 2015;99(9):1946-1952.

24. Hodge G, Hodge S, Chambers D, Reynolds PN, Holmes M. Bronchiolitis obliterans syndrome is associated with absence of suppression of peripheral blood Th1 proinflammatory cytokines. *Transplantation.* 2009;88(2):211-218.

25. Hodge G, Hodge S, Yeo A, et al. BOS Is Associated With Increased Cytotoxic Proinflammatory CD8 T, NKT-Like, and NK Cells in the Small Airways. *Transplantation.* 2017;101(10):2469-2476.

26. Hodge G, Hodge S, Yeo A, et al. BOS is associated with decreased HDAC2 from steroid resistant lymphocytes in the small airways. *Clin Exp Immunol.* 2019;195(2):277-285.

27. Hodge G, Hodge S, Liu H, Nguyen P, Holmes-Liew CL, Holmes M. Bronchiolitis obliterans syndrome is associated with increased senescent lymphocytes in the small airways. *J Heart Lung Transplant.* 2021;40(2):108-119.

28. Hodge S, Holmes M, Banerjee B, et al. Posttransplant bronchiolitis obliterans syndrome is associated with bronchial epithelial to mesenchymal transition. *Am J Transplant.* 2009;9(4):727-733.

29. Hodge S, Dean M, Hodge G, Holmes M, Reynolds PN. Decreased efferocytosis and mannose binding lectin in the airway in bronchiolitis obliterans syndrome. *J Heart Lung Transplant.* 2011;30(5):589-595.

30. Hübner RH, Meffert S, Mundt U, et al. Matrix metalloproteinase-9 in bronchiolitis obliterans syndrome after lung transplantation. *Eur Respir J.* 2005;25(3):494-501.

31. Ionescu DN, Girnita AL, Zeevi A, et al. C4d deposition in lung allografts is associated with circulating anti-HLA alloantibody. *Transpl Immunol.* 2005;15(1):63-68.

32. Kaes J, Van der Borght E, Vanstapel A, et al. Peripheral Blood Eosinophilia Is Associated with Poor Outcome Post-Lung Transplantation. *Cells.* 2020;9(11).

33. Keane MP, Gomperts BN, Weigt S, et al. IL-13 is pivotal in the fibro-obliterative process of bronchiolitis obliterans syndrome. *J Immunol.* 2007;178(1):511-519.

34. Krustrup D, Iversen M, Martinussen T, Schultz HH, Andersen CB. The number of FoxP3+ cells in transbronchial lung allograft biopsies does not predict bronchiolitis obliterans syndrome within the first five years after transplantation. *Clin Transplant.* 2015;29(3):179-184.

35. Laan M, Lindén A, Riise GC. IL-16 in the airways of lung allograft recipients with acute rejection or obliterative bronchiolitis. *Clin Exp Immunol.* 2003;133(2):290-296.

36. Leonard CT, Soccal PM, Singer L, et al. Dendritic cells and macrophages in lung allografts: A role in chronic rejection? *Am J Respir Crit Care Med.* 2000;161(4 Pt 1):1349-1354.

37. Magro CM, Ross P, Jr., Kelsey M, Waldman WJ, Pope-Harman A. Association of humoral immunity and bronchiolitis obliterans syndrome. *Am J Transplant.* 2003;3(9):1155-1166.

38. Magro CM, Pope Harman A, Klinger D, et al. Use of C4d as a diagnostic adjunct in lung allograft biopsies. *Am J Transplant.* 2003;3(9):1143-1154.

39. Magro CM, Abbas AE, Seilstad K, Pope-Harman AL, Nadasdy T, Ross P, Jr. C3d and the septal microvasculature as a predictor of chronic lung allograft dysfunction. *Hum Immunol.* 2006;67(4-5):274-283.

40. Mamessier E, Lorec AM, Thomas P, Badier M, Magnan A, Reynaud-Gaubert M. T regulatory cells in stable posttransplant bronchiolitis obliterans syndrome. *Transplantation.* 2007;84(7):908-916.

41. Meloni F, Vitulo P, Cascina A, et al. Bronchoalveolar lavage cytokine profile in a cohort of lung transplant recipients: a predictive role of interleukin-12 with respect to onset of bronchiolitis obliterans syndrome. *J Heart Lung Transplant.* 2004;23(9):1053-1060.

42. Meloni F, Solari N, Miserere S, et al. Chemokine redundancy in BOS pathogenesis. A possible role also for the CC chemokines: MIP3-beta, MIP3-alpha, MDC and their specific receptors. *Transpl Immunol.* 2008;18(3):275-280.

43. Neujahr DC, Perez SD, Mohammed A, et al. Cumulative exposure to gamma interferon-dependent chemokines CXCL9 and CXCL10 correlates with worse outcome after lung transplant. *Am J Transplant.* 2012;12(2):438-446.

44. Neurohr C, Huppmann P, Samweber B, et al. Prognostic value of bronchoalveolar lavage neutrophilia in stable lung transplant recipients. *J Heart Lung Transplant.* 2009;28(5):468-474.

45. Ngo C, Danel C, Duong-Quy S, et al. C4d detection and histological patterns in the diagnosis of antibody-mediated rejection after lung transplantation: a single-centre study. *Histopathology.* 2019;74(7):988-996.

46. Ramirez AM, Nunley DR, Rojas M, Roman J. Activation of tissue remodeling precedes obliterative bronchiolitis in lung transplant recipients. *Biomarker Insights.* 2008;2008(3):351-359.

47. Reynaud-Gaubert M, Thomas P, Gregoire R, et al. Clinical utility of bronchoalveolar lavage cell phenotype analyses in the postoperative monitoring of lung transplant recipients. *Eur J Cardiothorac Surg.* 2002;21(1):60-66.

48. Reynaud-Gaubert M, Marin V, Thirion X, et al. Upregulation of chemokines in bronchoalveolar lavage fluid as a predictive marker of post-transplant airway obliteration. *J Heart Lung Transplant.* 2002;21(7):721-730.

49. Riise GC, Ericson P, Bozinovski S, Yoshihara S, Anderson GP, Lindén A. Increased net gelatinase but not serine protease activity in bronchiolitis obliterans syndrome. *J Heart Lung Transplant.* 2010;29(7):800-807.

50. Sacreas A, von der Thüsen JH, van den Bosch TPP, et al. The pleural mesothelium and transforming growth factor-β(1) pathways in restrictive allograft syndrome: A pre-clinical investigation. *J Heart Lung Transplant.* 2019;38(5):570-579.

51. Saito T, Takahashi H, Kaneda H, et al. Impact of cytokine expression in the pre-implanted donor lung on the development of chronic lung allograft dysfunction subtypes. *Am J Transplant.* 2013;13(12):3192-3201.

52. Saito T, Liu M, Binnie M, Martinu T, Sato M, Keshavjee S. Upregulation of alveolar neutrophil enzymes and long pentraxin-3 in human chronic lung allograft dysfunction subtypes. *The Journal of thoracic and cardiovascular surgery.* 2018;155(6):2774-2776.e2772.

53. Sato M, Hirayama S, Hwang DM, et al. The role of intrapulmonary de novo lymphoid tissue in obliterative bronchiolitis after lung transplantation. *J Immunol.* 2009;182(11):7307-7316.

54. Sato M, Hirayama S, Matsuda Y, et al. Stromal activation and formation of lymphoid-like stroma in chronic lung allograft dysfunction. *Transplantation.* 2011;91(12):1398-1405.

55. Scholma J, Slebos DJ, Boezen HM, et al. Eosinophilic granulocytes and interleukin-6 level in bronchoalveolar lavage fluid are associated with the development of obliterative bronchiolitis after lung transplantation. *Am J Respir Crit Care Med.* 2000;162(6):2221-2225.

56. Shino MY, Weigt SS, Li N, et al. CXCR3 ligands are associated with the continuum of diffuse alveolar damage to chronic lung allograft dysfunction. *Am J Respir Crit Care Med.* 2013;188(9):1117-1125.

57. Sinclair KA, Yerkovich ST, Hopkins PMA, et al. The autotaxin-lysophosphatidic acid pathway mediates mesenchymal cell recruitment and fibrotic contraction in lung transplant fibrosis. *J Heart Lung Transplant.* 2021;40(1):12-23.

58. Snell GI, Levvey BJ, Zheng L, et al. Interleukin-17 and airway inflammation: a longitudinal airway biopsy study after lung transplantation. *J Heart Lung Transplant.* 2007;26(7):669-674.

59. Suwara MI, Vanaudenaerde BM, Verleden SE, et al. Mechanistic differences between phenotypes of chronic lung allograft dysfunction after lung transplantation. *Transpl Int.* 2014;27(8):857-867.

60. Suzuki H, Lasbury ME, Fan L, et al. Role of complement activation in obliterative bronchiolitis post-lung transplantation. *J Immunol.* 2013;191(8):4431-4439.

61. Vanaudenaerde BM, De Vleeschauwer SI, Vos R, et al. The role of the IL23/IL17 axis in bronchiolitis obliterans syndrome after lung transplantation. *Am J Transplant.* 2008;8(9):1911-1920.

62. Vandermeulen E, Verleden SE, Ruttens D, et al. BAL neutrophilia in azithromycin-treated lung transplant recipients: Clinical significance. *Transpl Immunol.* 2015;33(1):37-44.

63. Vandermeulen E, Verleden SE, Bellon H, et al. Humoral immunity in phenotypes of chronic lung allograft dysfunction: A broncho-alveolar lavage fluid analysis. *Transpl Immunol.* 2016;38:27-32.

64. Vandermeulen E, Lammertyn E, Verleden SE, et al. Immunological diversity in phenotypes of chronic lung allograft dysfunction: a comprehensive immunohistochemical analysis. *Transpl Int.* 2017;30(2):134-143.

65. Verleden SE, Vos R, Mertens V, et al. Heterogeneity of chronic lung allograft dysfunction: insights from protein expression in broncho alveolar lavage. *J Heart Lung Transplant.* 2011;30(6):667-673.

66. Verleden SE, Ruttens D, Vandermeulen E, et al. Elevated bronchoalveolar lavage eosinophilia correlates with poor outcome after lung transplantation. *Transplantation.* 2014;97(1):83-89.

67. Verleden SE, Ruttens D, Vos R, et al. Differential cytokine, chemokine and growth factor expression in phenotypes of chronic lung allograft dysfunction. *Transplantation.* 2015;99(1):86-93.

68. Verleden SE, Ruttens D, Vandermeulen E, et al. Predictors of survival in restrictive chronic lung allograft dysfunction after lung transplantation. *J Heart Lung Transplant.* 2016;35(9):1078-1084.

69. Verleden SE, Martens A, Ordies S, et al. Immediate post-operative broncho-alveolar lavage IL-6 and IL-8 are associated with early outcomes after lung transplantation. *Clin Transplant.* 2018;32(4):e13219.

70. Vos R, Vanaudenaerde BM, De Vleeschauwer SI, et al. C-reactive protein in bronchoalveolar lavage fluid is associated with markers of airway inflammation after lung transplantation. *Transplant Proc.* 2009;41(8):3409-3413.

71. Ward C, Whitford H, Snell G, et al. Bronchoalveolar lavage macrophage and lymphocyte phenotypes in lung transplant recipients. *J Heart Lung Transplant.* 2001;20(10):1064-1074.

72. Westall GP, Snell GI, McLean C, Kotsimbos T, Williams T, Magro C. C3d and C4d deposition early after lung transplantation. *J Heart Lung Transplant.* 2008;27(7):722-728.

73. Yang JYC, Verleden SE, Zarinsefat A, et al. Cell-Free DNA and CXCL10 Derived from Bronchoalveolar Lavage Predict Lung Transplant Survival. *J Clin Med.* 2019;8(2).

74. Zheng L, Walters EH, Ward C, et al. Airway neutrophilia in stable and bronchiolitis obliterans syndrome patients following lung transplantation. *Thorax.* 2000;55(1):53-59.

75. Zheng L, Orsida B, Whitford H, et al. Longitudinal comparisons of lymphocytes and subtypes between airway wall and bronchoalveolar lavage after human lung transplantation. *Transplantation.* 2005;80(2):185-192.

76. Zheng L, Whitford HM, Orsida B, et al. The dynamics and associations of airway neutrophilia post lung transplantation. *Am J Transplant.* 2006;6(3):599-608.
